# In Vitro and In Silico of Cholinesterases Inhibition and In Vitro and In Vivo Anti-Melanoma Activity Investigations of Extracts Obtained from Selected *Berberis* Species

**DOI:** 10.3390/molecules29051048

**Published:** 2024-02-28

**Authors:** Tomasz Tuzimski, Anna Petruczynik, Barbara Kaproń, Tomasz Plech, Anna Makuch-Kocka, Daria Janiszewska, Mateusz Sugajski, Bogusław Buszewski, Małgorzata Szultka-Młyńska

**Affiliations:** 1 Department of Physical Chemistry, Faculty of Pharmacy, Medical University of Lublin, Chodźki 4a, 20-093 Lublin, Poland; 2 Department of Inorganic Chemistry, Faculty of Pharmacy, Medical University of Lublin, Chodźki 4a, 20-093 Lublin, Poland; 3 Department of Clinical Genetics, Faculty of Medicine, Medical University of Lublin, Radziwiłłowska 11, 20-080 Lublin, Poland; barbara.kapron@umlub.pl (B.K.); tomasz.plech@umlub.pl (T.P.); anna.makuch-kocka@umlub.pl (A.M.-K.); 4 Department of Pharmacology, Faculty of Health Sciences, Medical University of Lublin, Radziwiłłowska 11, 20-080 Lublin, Poland; 5 Department of Environmental Chemistry and Bioanalytics, Faculty of Chemistry, Nicolaus Copernicus University, Gagarin 7, 87-100 Torun, Poland; janiszewska_daria@doktorant.umk.pl (D.J.); mateusz.sugajski@o2.pl (M.S.); bbusz@chem.umk.pl (B.B.); szultka.malgorzata@wp.pl (M.S.-M.)

**Keywords:** inhibition of cholinesterase activity, molecular docking, cytotoxic activity, *Danio rerio* larvae xenograft model, HPLC-DAD, LC-MS/MS, *Berberis* species

## Abstract

*Berberis* species have a long history of use in traditional Chinese medicine, Ayurvedic medicine, and Western herbal medicine. The aim of this study was the quantification of the main isoquinoline alkaloids in extracts obtained from various *Berberis* species by HPLC, in vitro and in silico determination of anti-cholinesterase activity, and in vitro and in vivo investigations of the cytotoxic activity of the investigated plant extracts and alkaloid standards. In particular, *Berberis* species whose activity had not been previously investigated were selected for the study. In the most investigated *Berberis* extracts, a high content of berberine and palmatine was determined. Alkaloid standards and most of the investigated plant extracts exhibit significant anti-cholinesterase activity. Molecular docking results confirmed that both alkaloids are more favourable for forming complexes with acetylcholinesterase compared to butyrylcholinesterase. The kinetic results obtained by HPLC-DAD indicated that berberine noncompetitively inhibited acetylcholinesterase, while butyrylcholinesterase was inhibited in a mixed mode. In turn, palmatine exhibited a mixed inhibition of acetylcholinesterase. The cytotoxic activity of berberine and palmatine standards and plant extracts were investigated against the human melanoma cell line (A375). The highest cytotoxicity was determined for extract obtained from *Berberis pruinosa* cortex. The cytotoxic properties of the extract were also determined in the in vivo investigations using the *Danio rerio* larvae xenograft model. The obtained results confirmed a significant effect of the *Berberis pruinosa* cortex extract on the number of cancer cells in a living organism. Our results showed that extracts obtained from *Berberis* species, especially the *Berberis pruinosa* cortex extract, can be recommended for further in vivo experiments in order to confirm the possibility of their application in the treatment of neurodegenerative diseases and human melanoma.

## 1. Introduction

Neurodegenerative diseases and cancer cause various public health problems due to the increase in disease cases and the deficit in effective therapies [1].

One of the most common neurodegenerative diseases is Alzheimer’s disease, which is characterized by a decline in memory and cognitive function in patients and is related to some adverse symptoms such as sleep disorders, loss of appetite, apathy, and depression [2].

The use of medicinal plants is an age-old tradition, and progress in modern medicine has caused the application of natural products worldwide for treating various diseases. Currently, the application of herbal drugs as complementary or alternative medicine is gaining popularity, and many drugs are simply extracted from plants, while others are chemically modified. Many medicinal plants show high activity to prevent, alleviate, and cure dangerous diseases such as neurodegenerative diseases and cancer.

Current medications used for treating neurodegenerative disorders can only reduce their symptoms. Most of the drugs that are currently used for treating Alzheimer’s disease (galanthamine, donezepil, and rivastigmine) are inhibitors of acetylcholinesterase. Acetylcholinesterase inhibitors bind to the enzyme and prevent its activity, which results in the accumulation of acetylcholine and thus causes a relief of the symptoms of Alzheimer’s disorder [2]. However, currently used acetylcholinesterase inhibitors are often associated with adverse effects such as weight loss, anorexia, and bradycardia. Therefore, there is a need to find new, more effective, and safer acetylcholinesterase inhibitors.

The genus *Berberis* is the most widely distributed natural origin of isoquinoline alkaloid berberine. Berberine displays diverse pharmacological activities such as antioxidant, antidiabetic, hypolipidemic, hepatoprotective, anti-inflammatory, antimicrobial, wound healing, antibacterial, antidepressant, and antitumour [3,4]. The alkaloids also have beneficial effects on neuropsychiatric and neurodegenerative disorders. Additionally, berberine exhibits good bioavailability because of the presence of a long-chain alkylation, which enhances its hydrophobicity. 

Various *Berberis* species contain high amounts of isoquinoline alkaloids, especially berberine, and are commonly used in traditional medicine. Several *Berberis* species exhibited activity against viruses, fungi, protozoa, helminths and chlamydia, antiarrhythmic, antibacterial, antirheumatic, anticholinergic, antihistaminic, antihypertensive, antinociceptive, and vasodilatory effects [5]. 

The inhibition of cholinesterase activity by extracts obtained from various *Berberis* species has been previously reported. For example, the extract obtained from *Berberis bealei* was tested as an acetylcholinesterase activity inhibitor using the modified Ellman method [6]. The highest activity was exhibited by dichloromethane extract (IC_50_ = 9.99 μg/mL), while the IC_50_ value obtained for galantamine was 4.33 μg/mL. Hostalkova et al. determined the anticholinesterase activity of alkaloids isolated from *Berberis vulgaris* root bark extract [7]. The most active against acetylcholinesterase was berberine, with IC_50_ = 0.7 µM, while the highest inhibitory activity against butyrylcholinesterase was observed for aromoline (IC_50_ = 0.82 µM). Butyrylcholinesterase was inhibited by the most active n-hexane fractions of *Berberis libanotica* and *Berberis aetnensis,* with IC_50_ = 47.0 and 78.1 μg/mL, respectively. Extracts obtained from various *Berberis* species also exhibited cytotoxic properties. The cytotoxic activity of total alkaloids extracted from *Berberis hispanica* against human laryngeal carcinoma cells (Hep-2 cell line), with IC_50_ = 75 μg/mL, was reported [8]. The extract obtained from *Berberis thunbergii* leaf showed cytotoxic activity against pancreatic (PANC-1, AsPC-1, and MIA PaCa-2) cell lines, with IC_50_ = 259, 268, and 141 μg/mL, respectively [9]. In another study, the cytotoxic activity of *Berberis vulgaris* roots extract showed cytotoxicity against breast (MCF7), liver (HepG2) and colon (CACO-2) cancer cell lines [10]. All tested cells’ viability was significantly decreased in a dose-dependent manner after treatment with both berberine chloride and *Berberis vulgaris* ethanolic extracts. The cytotoxic properties of the extract obtained from *Berberis aristata* root bark were tested against human rhabdomyosarcoma (RD), diploid mouse lung cell line (L20B), and human larynx epidermoid carcinoma (Hep 2) cell lines [11]. The cytotoxicity was observed against all tested cell lines, with IC_50_ values ranging from 245 to 473 μg/mL. Bersavine, a bisbenzylisoquinoline alkaloid isolated from *Berberis vulgaris,* inhibits the proliferation and viability of leukemic (Jurkat, MOLT-4), colon (HT-29), cervix (HeLa), and breast (MCF-7) cancer cells, with IC_50_ values ranging from 8.1 to 11 μM [12]. A relatively low cytotoxicity (compared to doxorubicin) was reported for *Berberis integerrima* leaves extract against breast cancer (MCF-7), epidermal (A431), and glioma (U87-MG) cell lines [13].

Cancer cell viability was also tested on the cervical cancer cell line (HeLa) after treatment with a combination of berberine obtained from *Berberis vulgaris* root and cisplatine, a chemotherapeutic agent [14]. The obtained results indicated that the combination of berberine and cisplatin exhibits an additive effect on reduction in cancer cell growth.

The next step of anticancer activity exploration of potential candidates for drugs that exhibited the highest in vitro activity should be in vivo investigations using animal models. Nude mice are most commonly used for this type of experiment, but the research takes a long time and they are expensive. To overcome these shortcomings, an alternative animal model using the *Danio rerio* larvae is increasingly being used to test potential anti-cancer drugs [15]. The *Danio rerio* larvae model has numerous advantages, e.g., the ability to study a large number of individuals simultaneously, a shorter duration of the experiments, and a large number of experiments that can be carried out at affordable costs. 

To the best of our knowledge, the cytotoxic activity of *Berberis* species extracts using the *Danio rerio* larvae xenograft model has not been investigated previously.

Numerous reports suggest that extracts obtained from different *Berberis* species show various activities and therefore require further research in order to select species with the highest activity, which could be potentially used in the treatment of different diseases. 

In the present study, we determined the acetylcholinesterase and butyrylcholinesterase inhibition activity of alkaloid standards and various *Berberis* species extracts by the HPLC-DAD method. The HPLC-DAD was also applied for a kinetics study performed for berberine and palmatine. The interaction of berberine and palmatine with both cholinesterases was also investigated by in silico docking simulations, and binding affinities and binding modes were determined. The aim of this work was also in vitro and in vivo determination of the anti-melanoma activity of isoquinoline alkaloid standards and plant extracts prepared from various Berberis species. In in vitro investigations, the viability of human melanoma cells (A375) treated by alkaloid standards and plant extracts was determined and compared with the cytotoxicity of the anticancer drug cisplatin. Cytotoxicity of extract obtained from the cortex of *Berberis pruinosa* exhibited high cytotoxic properties and was investigated in vivo using *Danio rerio* larvae xenograft experiments. Our investigations may be the basis for further in-depth studies of *Berberis* species extracts in terms of their development as cholinesterase inhibitors and anti-cancer agents for the treatment of melanoma.

## 2. Results and Discussion

### 2.1. Determination of Alkaloid Contents in Plant Extracts

Berberine and palmatine were analysed by HPLC-DAD on a Polar RP column with a mobile phase composed of acetonitrile, water, and 0.05 ML^−1^ of 1-butyl-3-methylimidazolium tetrafluoroborate using the gradient system described in section “Experimental”. The applied chromatographic system was based on the previously published procedure after appropriate modification [16].

**Figure 1:**
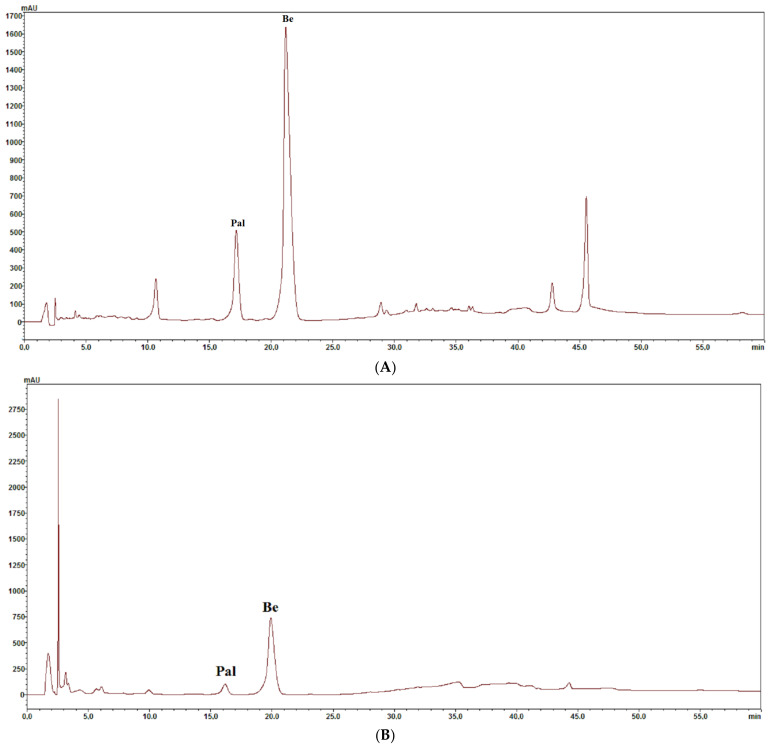
(**A**) Chromatogram obtained for the extract from the cortex of *Berberis pruinosa*. Pal: palmatine, Be: berberine. (**B**) Chromatogram obtained for extract from fruits of *Berberis thumbergii*. Pal: palmatine, Be: berberine.

The same chromatographic system was applied for the analysis of alkaloids in plant extracts obtained from the cortex and fruits of *Berberis thunbergii*, the cortex of *Berberis pruinosa*, *Berberis gagnepainii*, *Berberis veitchii*, *Berberis candidula*, and *Berberis aquifolium*. The confirmation of alkaloid presence in investigated extracts was performed by comparison of retention times, UV–Vis spectra, and MS spectra. An exemplary chromatogram obtained for *Berberis pruinosa* cortex extract is presented in Figure 1A, and a chromatogram obtained for *Berberis thunbergii* fruit extract is presented in Figure 1B.

The determination of alkaloid content in plant extracts was performed by a calibration curve method. The equations of the calibration curves obtained for berberine and palmatine, the correlation coefficient (r), the limit of detection (LOD) and the limit of quantification (LOQ) are presented in Table 1.

The average content of both investigated alkaloids was significantly different in extracts obtained from various *Berberis* species (Table 2). The extract obtained from the cortex of *Berberis pruinosa* contained the highest amount of berberine (1.150 mg/g of dry plant material). A high content of alkaloids was also found in the extract from the cortex of *Berberis thunbergii* (0.919 mg/g of dry plant material). The lowest content of berberine (0.058 mg/g of dry plant material) was determined in *Berberis gagnepainii* cortex extract. In the extract, the lowest amount of palmatine was also observed (0.034 mg/g of dry plant material). The highest content of palmatine was determined in the extract from the cortex of *Berberis aquifolium* (0.550 mg/g of dry plant material). Significant differences in the content of alkaloids were also found in extracts obtained from the cortex and fruits of *Berberis thunbergii*. In the extract from the cortex, a higher content of berberine was determined (0.919 and 0.212 mg/g of dry plant material in the cortex and fruit extracts, respectively), while a higher amount of palmatine was found in extracts from fruits (0.055 and 0.139 mg/g of dry plant material in the cortex and fruit extracts, respectively).

Various studies have demonstrated the presence of isoquinoline alkaloids in extracts obtained from different *Berberis* species. In a previous study, extracts obtained from various parts of *Berberis chitria*, *Berberis jaeschkeana*, *Berberis koehneana*, *Berberis lyceum*, *Berberis pseudoumbellata*, *Berberis aristata*, *Berberis asiatica* and *Berberis petiolaris* have been found from 0.002 to 89.2 mg of palmatine and from 0.91 to 172.8 mg of berberine in g of dry plant materials [17]. In *Berberis jaeschkeana* root extract, 46.38  mg/g of berberine and 20.54  mg/g of palmatine have been determined [18]. Our results of the chromatographic analysis of berberine and palmatine contents in tested extracts showed high concentrations of these alkaloids in the most investigated extracts. The highest content of berberine was determined in *Berberis pruinosa* cortex extract (1.150 mg/g of dry plant material), and the highest content of palmatine was observed in the extract obtained from the cortex of *Berberis aquifolium* (0.550 mg/g of dry plant material). Quantification of berberine and palmatine in the extract obtained from *Berberis pruinosa* was performed for the first time. 

### 2.2. LC-QqQ-ESI-MS/MS for the Identification of Selected Alkaloids in Plant Extracts

**Figure 2:**
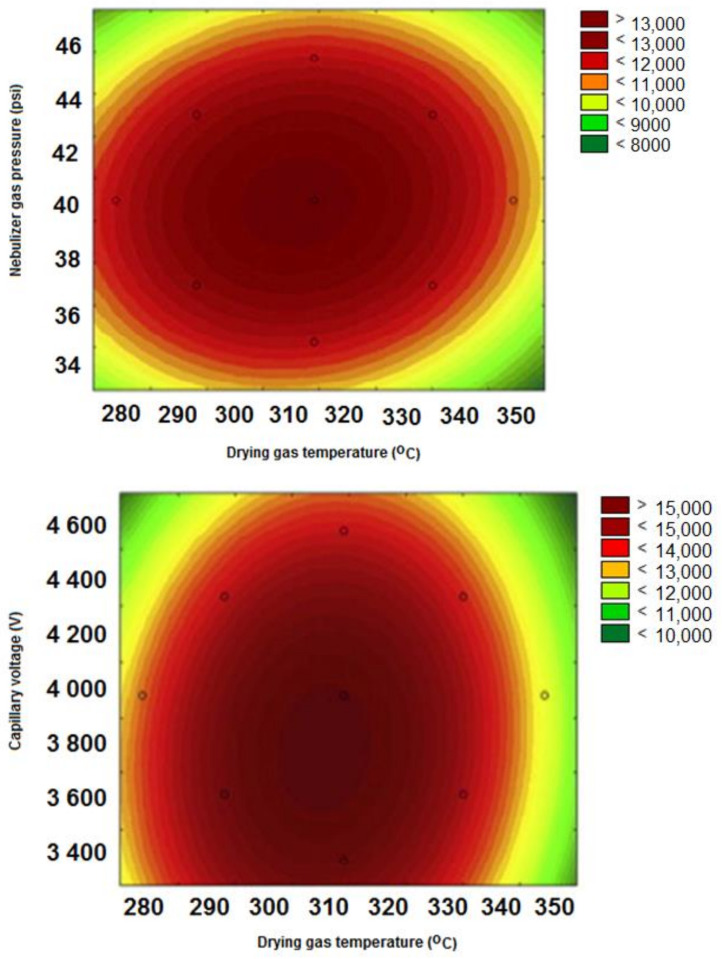

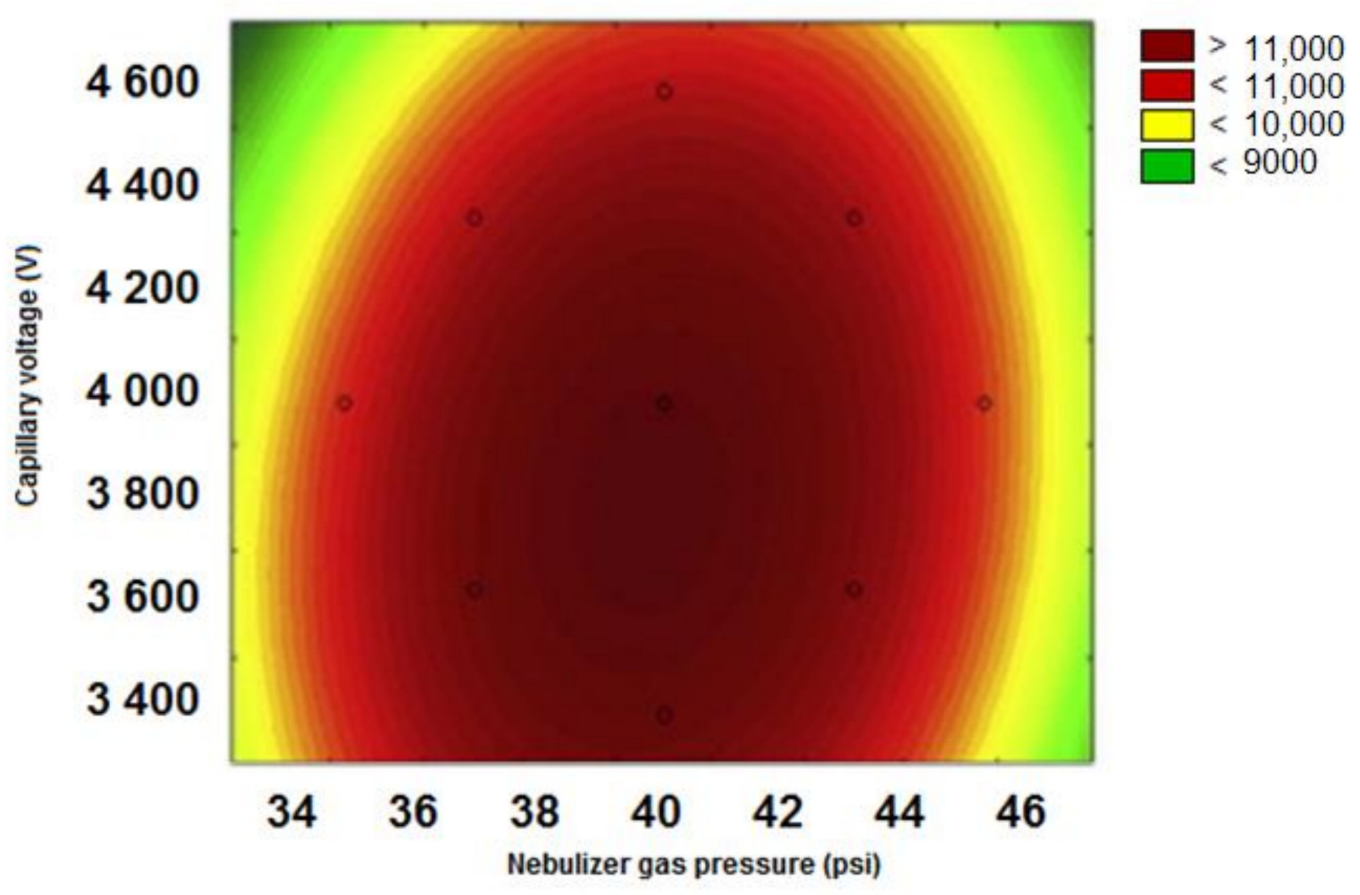
3D surface for the MS measurements with the use of the CCD approach. Response surface plots showing the effects of drying gas temperature, nebulizer gas pressure and capillary voltage on the intensity of berberine (model compound).

For the optimization step, Statistica 13.1 software designed 16 experiments in accordance with the Central Composite Design (CCD). The obtained experimental results are summarized in Table 3. A second-order polynomial model was constructed, where Y is the predicted response value and X1 (drying gas temperature), X2 (nebulizer gas pressure), and X3 (capillary voltage) are the coded values. The typical 3D response surface plots for berberine are shown in Figure 2. Based on the plotted surfaces, the response value reached a maximum when X1, X2, and X3 were around the central points. The LC-MS analysis conditions for the berberine (X1 = 350 °C, X2 = 35 psi, X3 = 4000 V) were obtained (Table 3). Factors and their levels with the design matrix for the 2^3^ Central Composite Design (CCD).

**Figure 3:**
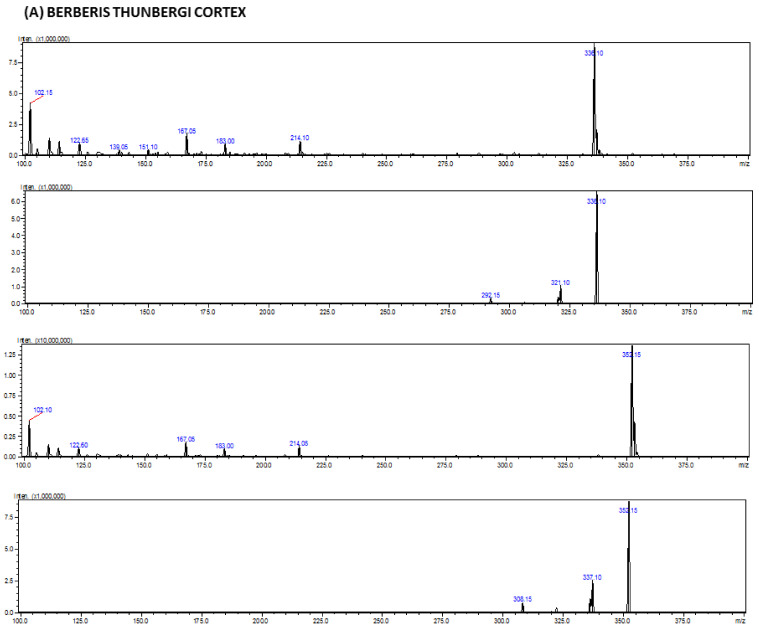

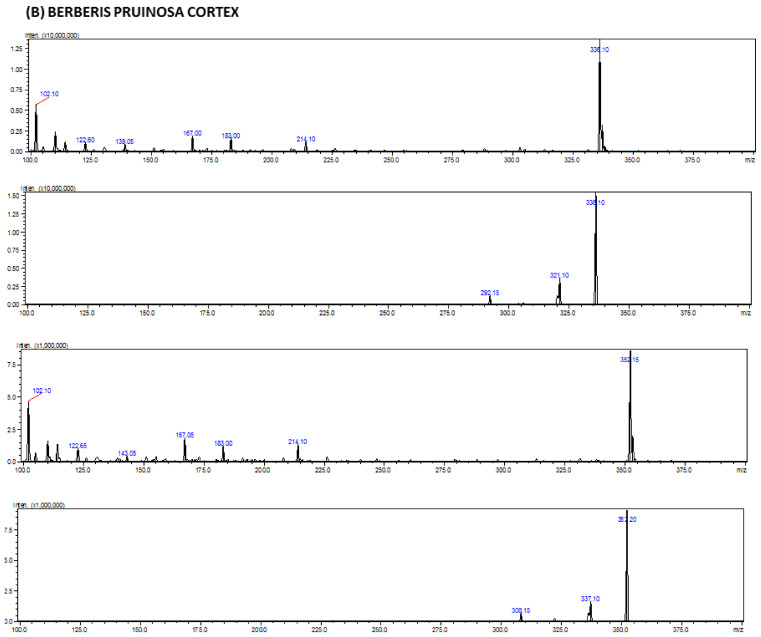

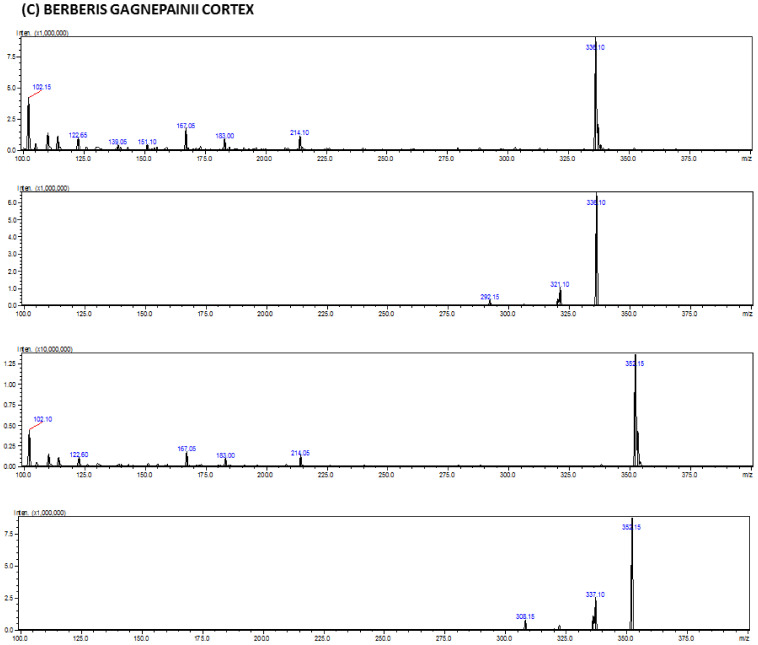

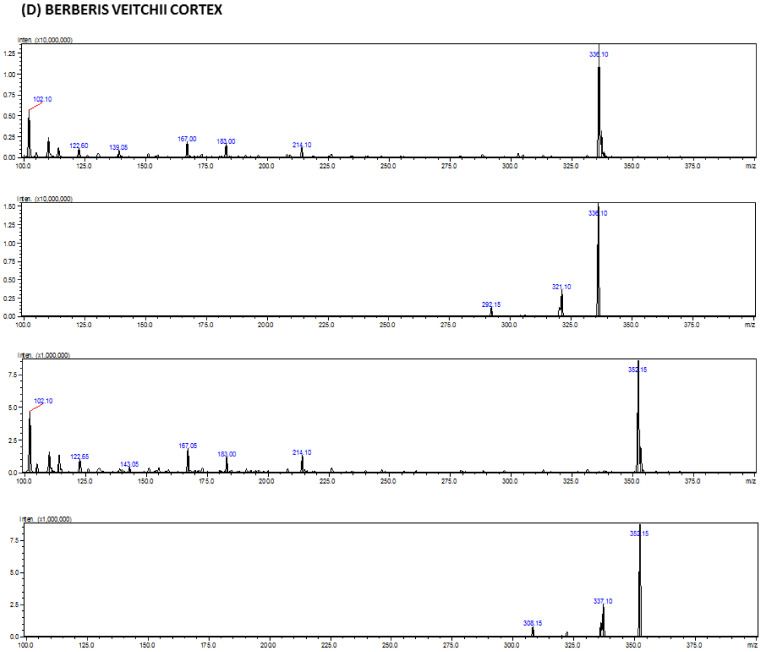

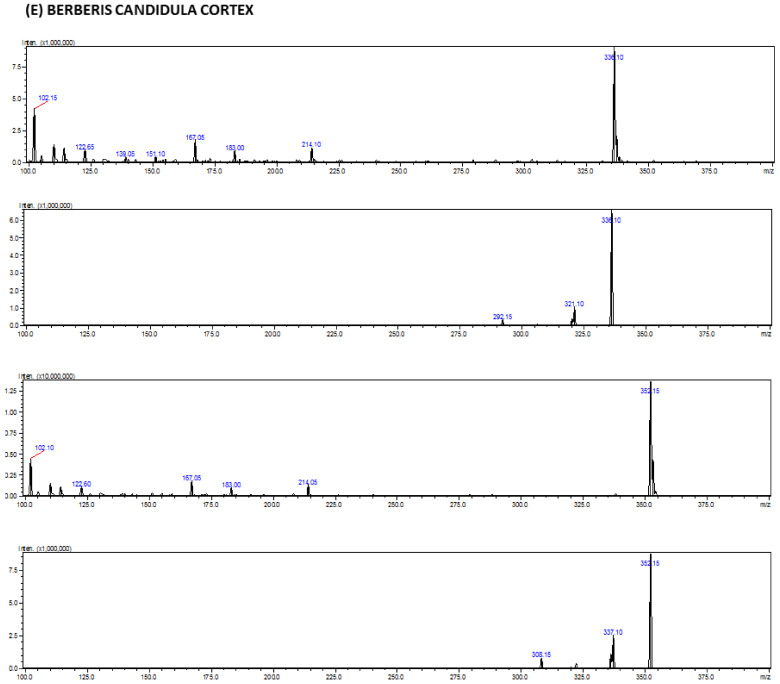

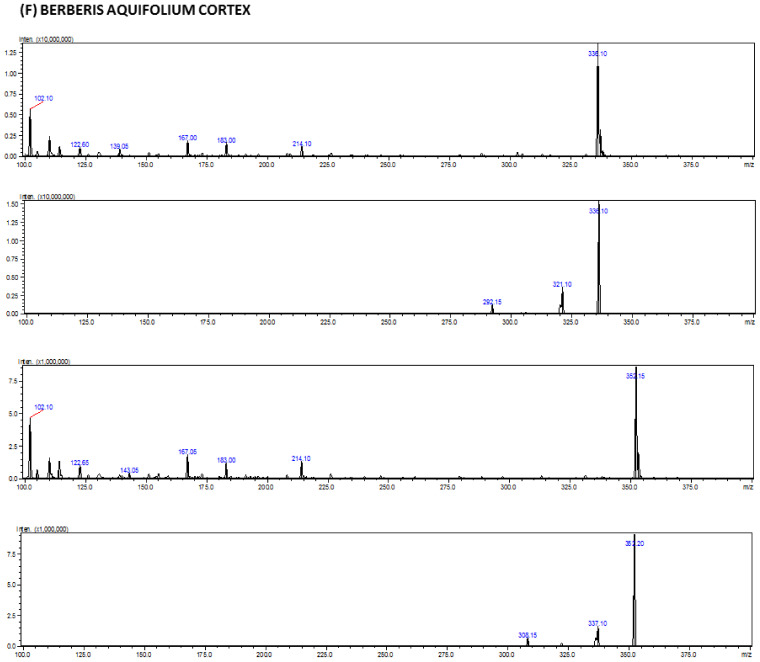

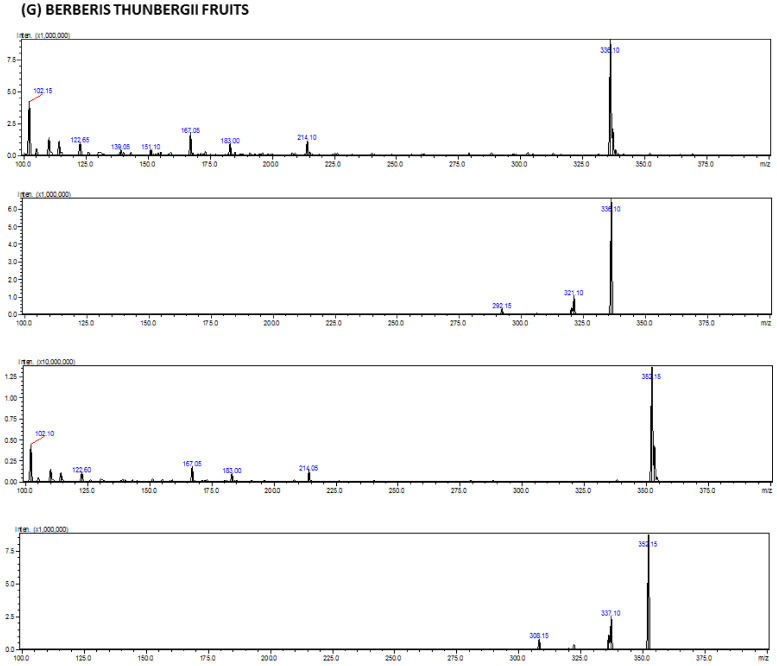
Representative MS spectra for berberine (cortex, fruits) obtained for the studied isoquinoline alkaloid from *Berberis* extracts. (**A**) *Berberis thunbergi* cortex; (**B**) *Berberis pruinosa* cortex; (**C**) *Berberis gagnepainii* cortex; (**D**) *Berberis veitchii* cortex; (**E**) *Berberis candidula* cortex; (**F**) *Berberis aquifolium* cortex; (**G**) *Berberis thunbergia* fruits.

The proof of the identity of the berberine in real samples was confirmed by the comparison of its retention times and UV spectra with the retention times, as well as the MS spectra and product ion MS/MS fragmentation pattern. Isoquinoline alkaloid was quantified in extracts obtained from various parts (cortex, fruits) of Berberis extracts. It was identified based on MS spectra for berberine (*m*/*z* = 335.15). Representative MS spectra obtained for alkaloids from extracts are presented in Figure 3.

**Figure 4:**
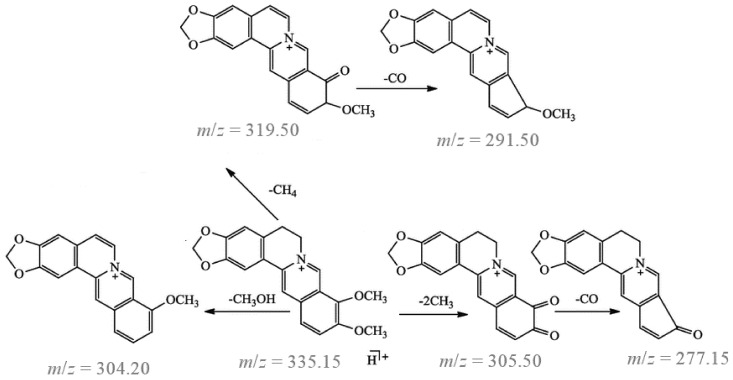
MS/MS fragmentation pattern for berberine.

Figure 4 shows the MS/MS fragmentation pattern for the target compound. The major product ion is at *m*/*z* = 319.50 and corresponds to the elimination of the methyl radical and CH_4_ group from the methoxy substituent. The ion at *m*/*z* = 305.50 was created by the continuous elimination of two methyl radicals. The ion at *m*/*z* = 304.20 was created by the loss of CH_3_OH from the precursor ion. The ions at *m*/*z* = 291.50 and 277.15 were created by the loss of CO from the ions at *m*/*z* = 319.15 and *m*/*z* = 305.50, respectively. 

### 2.3. Determination of Cholinesterases Inhibition Activity of Alkaloid Standards

For in vitro determination of cholinesterase activity inhibition by alkaloid standards, the Ellman method with HPLC-DAD was applied. Both investigated alkaloids showed high absorption at λ = 412 nm. The same wavelength was used for the determination of 5-thio-2-nitro-benzoic acid, a product reaction of thiocholine hydrolysis by acetyl/butyrylthiocholine with 5,5′-dithiobis-2-nitrobenzoic acid. For this reason, the determination of cholinesterase activity inhibition by the chromatographic method is more precise compared to the commonly used spectrophotometric method. Galantamine and rivastigmine were used in our experiments as a positive control. The IC50 values for both alkaloids were calculated for concentrations in the range of 0.1–200 µM. The obtained results are presented in Table 4. For all investigated alkaloids higher activity against acetylcholinesterase compared to anti-butyrylcholinesterase activity was observed. Berberine exhibited higher cholinesterase inhibition activity than palmatine, with IC_50_ = 2.04 μM (0.69 μg/mL) against acetylcholinesterase and 12.42 μM (4.18 μg/mL) against butyrylcholinesterase. The IC_50_ values of 9.14 and 189.40 μM were obtained for palmatine against acetylcholinesterase and butyrylcholinesterase, respectively. These results confirmed previously reported higher berberine cholinesterase inhibition activity compared to the activity of palmatine (IC_50_ values against acetylcholinesterase of 2.2 and 7.4 μg/mL obtained for berberine and palmatine, respectively, and against butyrylcholinesterase of 105.1 and 116.7 μg/mL obtained for berberine and palmatine, respectively) [5]. Berberine showed very high inhibition of acetylcholinesterase activity. The IC_50_ values determined for berberine were slightly higher than the IC_50_ values obtained in the same conditions for galantamine (1.46 μM) and lower than the IC_50_ value obtained for rivastigmine (10.23 μM) [19]. Palmatine showed moderate inhibition of acetylcholinesterase activity and low activity against butyrylcholinesterase. 

In previous studies, it was found that berberine and palmatine exhibited acetylcholinesterase inhibition activity. For example, Bonesi et al. reported that berberine and palmatine inhibited acetylcholinesterase activity with IC_50_ values of 2.2 and 7.4 μg/mL, respectively, and butyrylcholinesterase activity with IC_50_ values of 105.1 and 116.7 μg/mL, respectively [5]. Results obtained in our investigations confirmed previously described higher acetylcholinesterase inhibition activity of berberine compared to palmatine, with IC_50_ = 2.04 μM (0.69 μg/mL) against acetylcholinesterase and 12.42 μM (4.18 μg/mL) against butyrylcholinesterase. The IC_50_ values of 9.14 and 189.40 μM were obtained for palmatine against acetylcholinesterase and butyrylcholinesterase, respectively.

### 2.4. Inhibition of Cholinesterases Activity by Plant Extracts

In vitro cholinesterase inhibition activity by extracts obtained from various *Berberidaceae* species was also determined by the Ellman method with HPLC-DAD. Extracts obtained from various *Berberidaceae* species exhibited significantly different anticholinesterase activity (Table 5). Most of the investigated extracts, except the extract obtained from the cortex of *Berberis gagnepainii,* significantly inhibited both acetylcholinesterase and butyrylcholinesterase activity. Differences in activity were also observed for extracts obtained from the cortex and fruits of *Berberis thunbergii*. The highest anti-acetylcholinesterase activity was found for the extract obtained from the cortex of *Berberis pruinosa* (IC_50_ = 5.52 μg/mL). A similar anti-acetylcholinesterase activity was found in the extract obtained from the cortex of *Berberis aquifolium*. The IC_50_ value of 5.76 μg/mL was determined for the extract. High activity against acetylcholinesterase was also observed for extracts obtained from the cortex of *Berberis thunbergii*, *Berberis candidula,* and *Berberis veitchii* (IC_50_ = 11.02, 18.46, and 18.60 μg/mL, respectively). 

The highest inhibition of butyrylcholinesterase activity was determined for the extract from the cortex of *Berberis aquifolium* (IC_50_ = 10.45 μg/mL). Significantly anti-butyrylcholinesterase activity was also observed for extracts obtained from the cortex of *Berberis thunbergii* and *Berberis veitchii* (IC_50_ = 26.12 and 40.40 μg/mL, respectively). 

In most cases, the anti-acetylcholinesterase activity of investigated extracts was higher compared to their activity against butyrylcholinesterase. For example, the extract obtained from *Berberis thunbergii* cortex inhibited acetylcholinesterase activity with IC_50_ = 11.02 μg/mL, while the same extract inhibited butyrylcholinesterase with IC_50_ = 26.12 μg/mL. 

To the best of our knowledge, all investigated extracts obtained from *Berberis* species, except extracts from *Berberis thunbergii* (but obtained from leaves) [20], were not previously investigated for their acetylcholinesterase inhibition activity. Some publications describe the anti-neurodegenerative diseases activity of extracts obtained from other *Berberis* species. For example, extracts obtained from the roots of *Berberis aetnensis,* C. Presl., and *Berberis libanotica* Ehrenb. ex C.K. Schneid. exhibited anti-acetylcholinesterase activity, with IC_50_ values of 7.6 μg/mL and 16.9 μg/mL, respectively [5].

### 2.5. Kinetic Study of the Cholinesterases Inhibition

**Figure 5:**
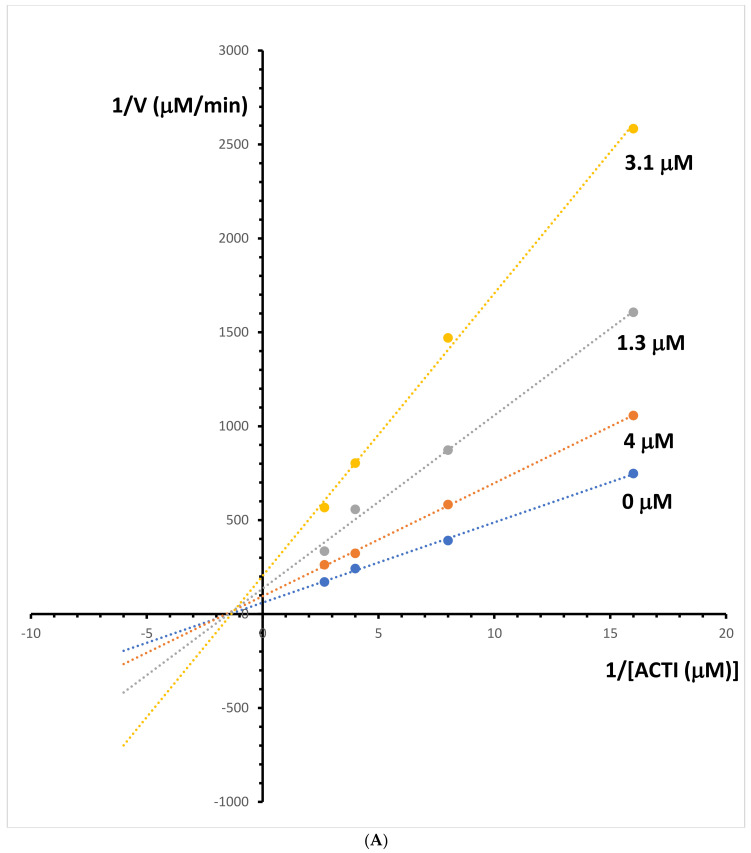

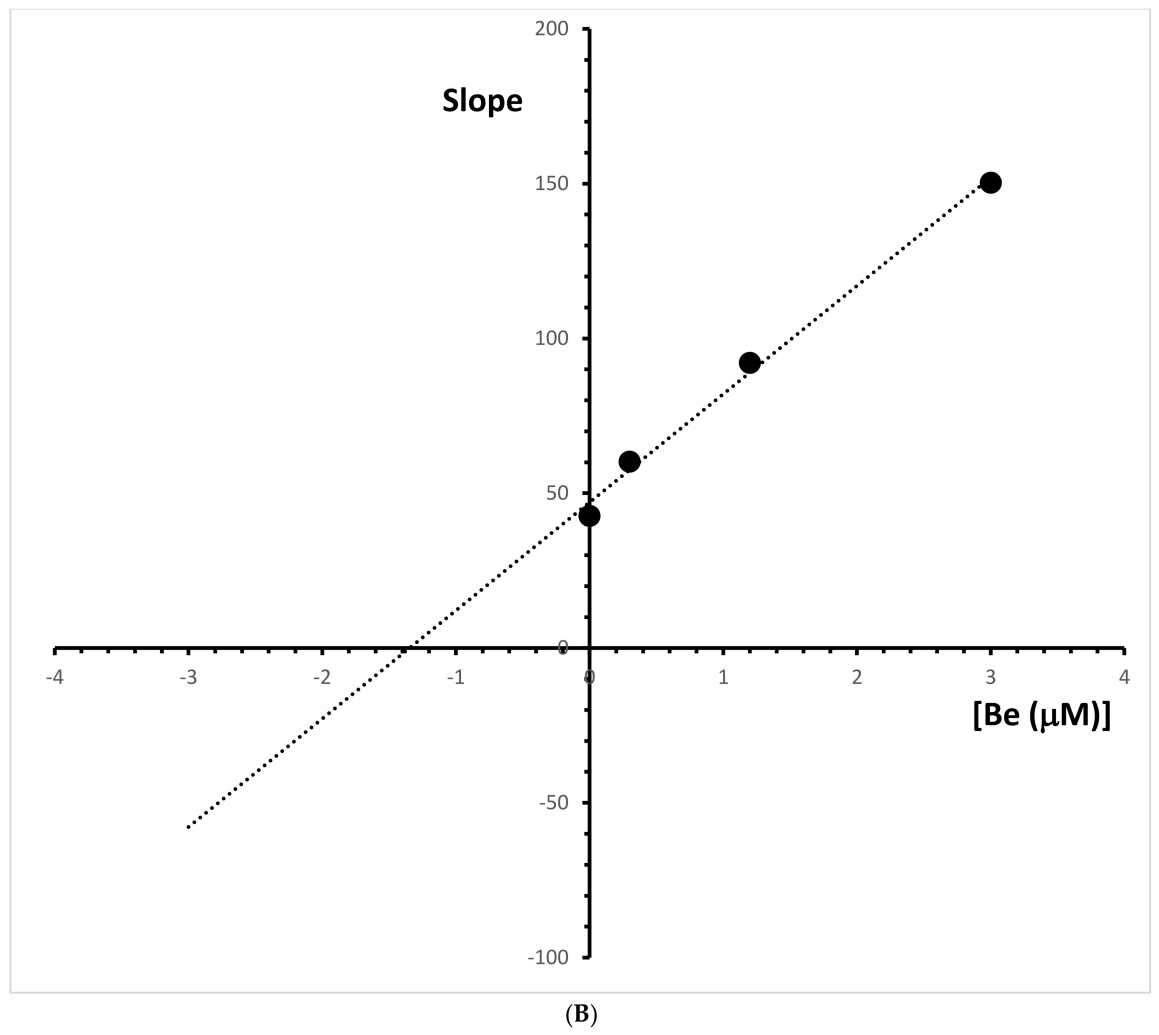
(**A**) Lineweaver–Bulk plot of acetylcholinesterase activity over a range of substrate-acetylthiocholine iodide (ACTI) concentration for berberine. (**B**) Determination of acetylcholinesterase inhibition constants (Ki) by plotting the slope of the primary Lineweaver–Burk plot vs. berberine (Be) concentration.

**Figure 6:**
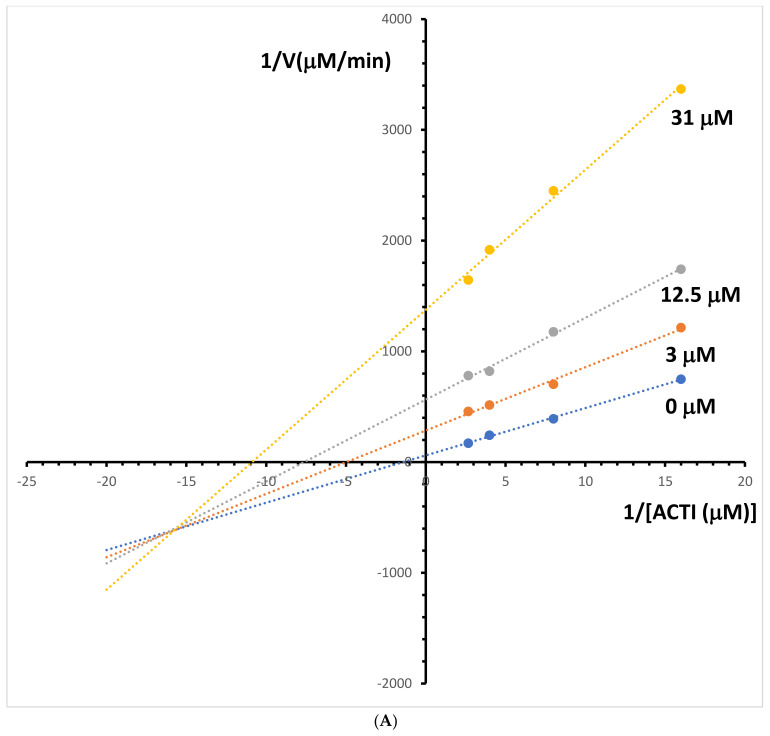

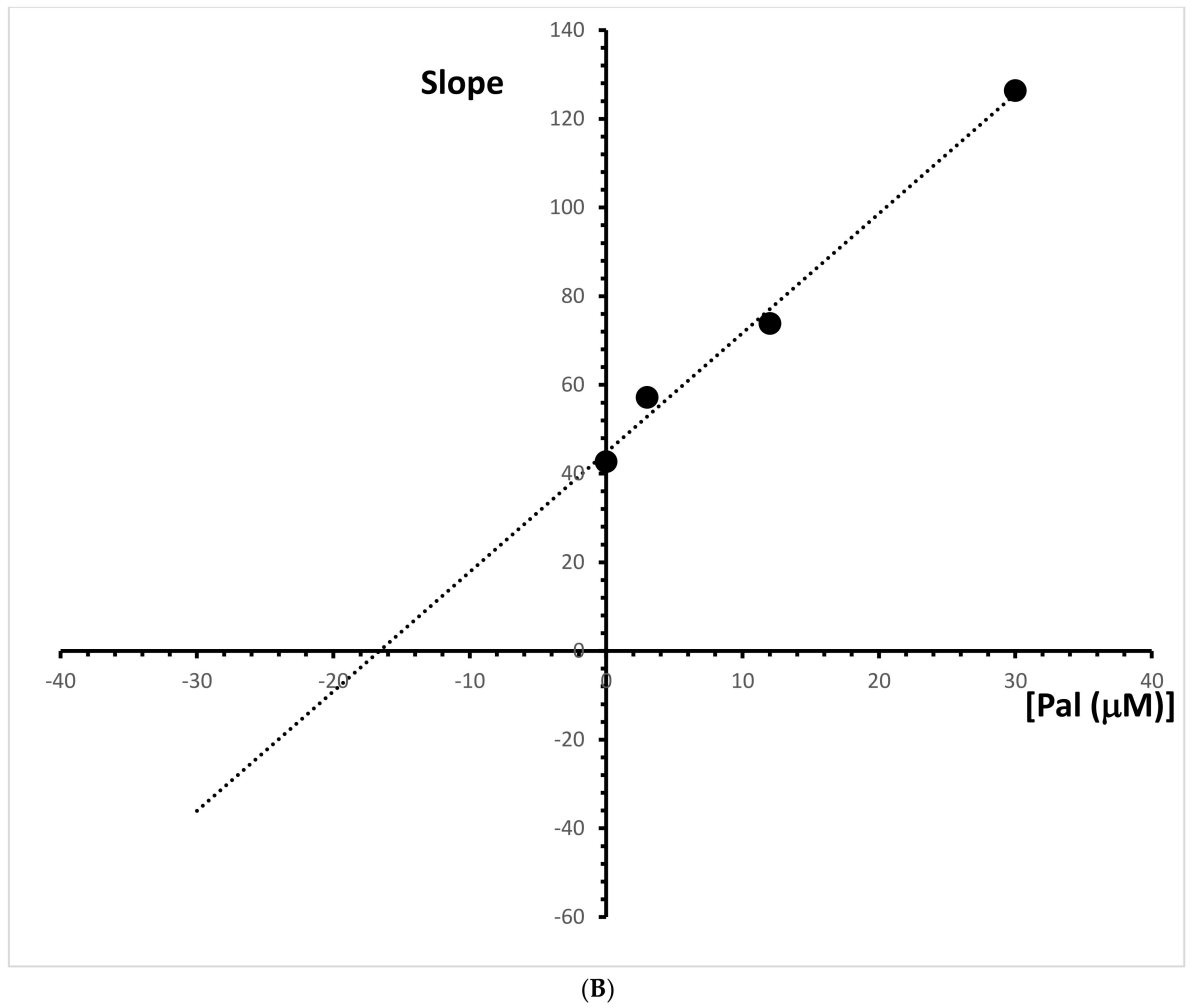
(**A**) Lineweaver–Bulk plot of acetylcholinesterase activity over a range of substrate-acetylthiocholine iodide (ACTI) concentration for palmatine. (**B**) Determination of acetylcholinesterase inhibition constants (Ki) by plotting the slope of the primary Lineweaver–Burk plot vs. palmatine (Pal) concentration.

The enzyme kinetic study was performed for both investigated alkaloids: berberine and palmatine. Lineweaver–Burk and Dixon plots were used for the determination of the type of inhibition and inhibition constants (Ki value) of these alkaloids. The Lineweaver–Burk plots obtained at different acetylcholinesterase inhibitor concentrations for berberine are shown in Figure 5A. The resulting plots showed that berberine displayed a noncompetitive inhibition behaviour. The obtained results confirm the previously reported non-competitive inhibition mode for this alkaloid [21]. The Ki value was estimated from the replots of the slope of the individual Lineweaver–Burk plots versus the inhibitor concentrations (Figure 5B). The Ki value of 1.4 μM for acetylcholinesterase inhibition by berberine was determined. The type of acetylcholinesterase inhibition and Ki value was determined for palmatine by the same method. The intersection of lines is located outside of the *y*- or *x*-axis, which suggests a mixed-type inhibition of acetylcholinesterase activity (Figure 6A). The mixed mode of inhibition for this alkaloid was also obtained in earlier studies using the spectrophotometric method [21]. The Ki value for palmatine was estimated at 17 μM (Figure 6B). 

**Figure 7:**
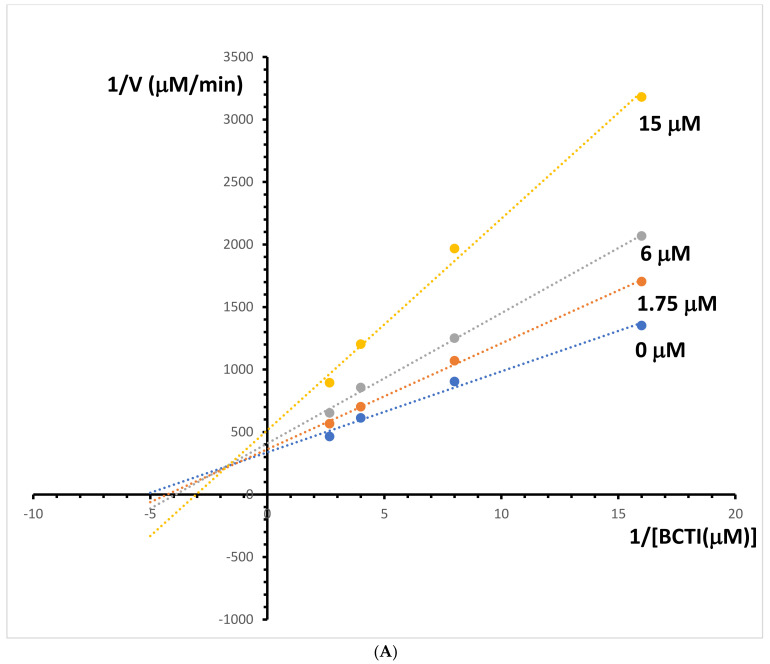

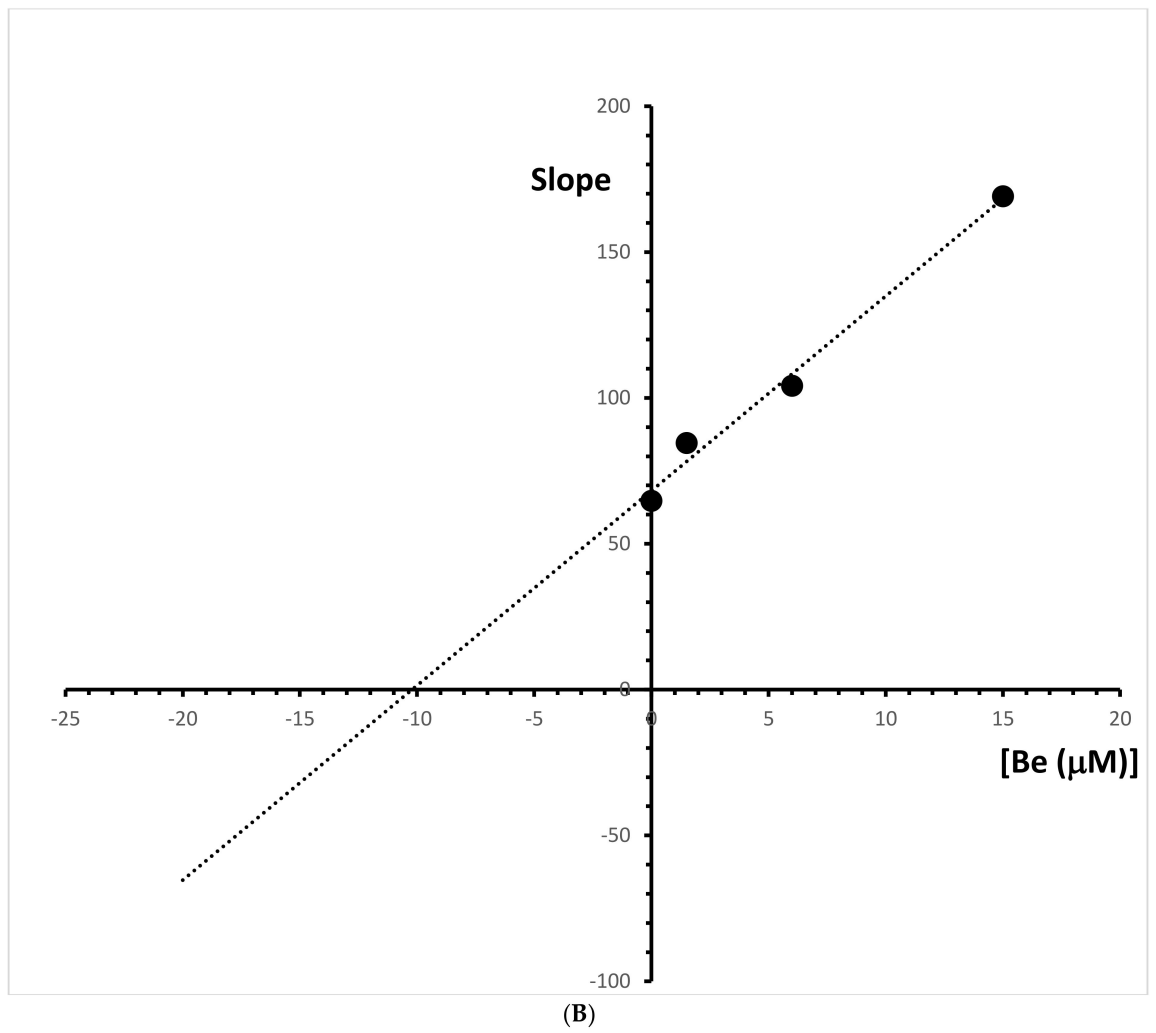
(**A**) Lineweaver–Bulk plot of butyrylcholinesterase activity over a range of substrate-butyrylthiocholine iodide (BCTI) concentration for berberine. (**B**) Determination of butyrylcholinesterase inhibition constants (Ki) by plotting the slope of the primary Lineweaver–Burk plot vs. berberine (Be) concentration.

Kinetic study was also performed for inhibition of butyrylcholinesterase activity by more active alkaloid berberine. The results presented in Figure 7A suggested that berberine is a mixed-mode inhibitor of butyrylcholinesterase. The Ki value of butyrylcholinesterase activity inhibition by the alkaloid was 10.5 μM, derived from the secondary replot of the Lineweaver–Burk plot (Figure 7B). Due to the low anti-butyrylcholinesterase activity of palmatine, no kinetic study has been performed.

The kinetic study indicated that berberine non-competitively inhibited acetylcholinesterase, which confirms the previously reported non-competitive inhibition mode for this [21]. The mixed-type inhibition of acetylcholinesterase activity was observed for palmatine. The mixed-mode of inhibition for this alkaloid was also obtained in earlier studies using the spectrophotometric method [21]. 

### 2.6. Molecular Docking

In our previous study, we described the methodology employed using the Molecular Operating Environment (version, MOE 2022.02) software (https://www.chemcomp.com/trial, accessed on 16 January 2024) to identify active sites in AChE and BChE proteins. Berberine and palmatine, obtained from the ZINC database as clean mol2 structures with assigned Gasteiger charges, were utilized as ligands in the investigation. Each analysis consisted of 100 runs, with a maximum evaluation limit set at 2,500,000. Once the maximum number of evaluations was reached, the current generation was terminated without initiating a new one.

**Figure 8:**
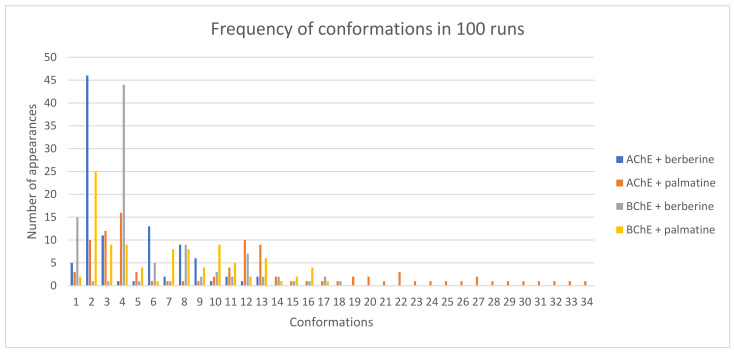
Frequency of conformations in 100 runs for all analysis.

**Figure 9:**
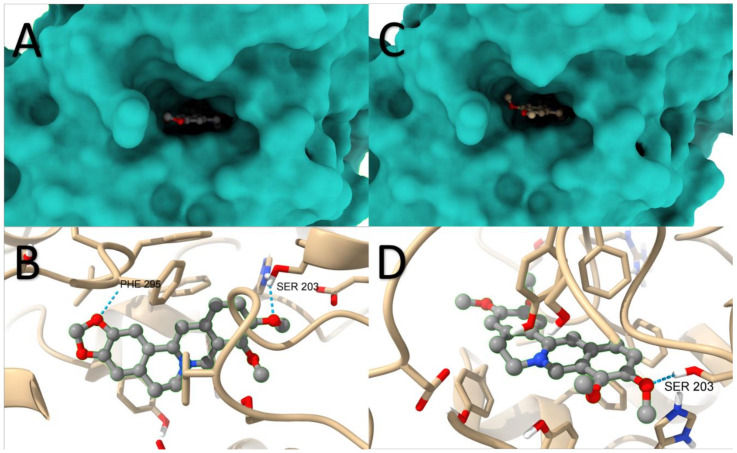
The location in AChE of berberine (**A**) and palmatine (**C**) along with the hydrogen bonds formed for berberine (**B**) and palmatine (**D**).

Docking experiments with AChE and berberine resulted in 100 conformations, and RMSD cluster analysis was conducted using all 25 ligand atoms (Figure 8). This analysis revealed the presence of 13 distinct conformational clusters among the 100 runs, with a standard RMSD tolerance of 2.0 Å. The corresponding graph illustrates the distribution of runs across each conformation, with run number 66 producing the most favourable outcome, exhibiting an estimated free binding energy of −9.30 kcal/mol. This energy value is derived from the summation of the final intermolecular energy (−9.90 kcal/mol), final total internal energy (−0.10 kcal/mol), torsional free energy (0.60 kcal/mol), and unbound system energy (−0.10 kcal/mol). The estimated inhibition constant (Ki) was determined as 152.66 nM. Additional analysis using the Protein-Ligand Interaction Profiler software (2023 online version) revealed the potential formation of five hydrogen bonds involving residues 121GLY, 122GLY, 203SER, 295PHE, and 447HIS. The image depicts the spatial arrangement of the ligand within the protein structure (Figure 9A) and highlights the hydrogen bond with the highest probability (Figure 9B).

By substituting berberine with palmatine, 100 conformations were also obtained, and RMSD cluster analysis was performed using 26 ligand atoms with a tolerance of 2.0 Å. The corresponding graph indicates the distribution of runs among the different conformations, totalling 34 in this case. Run number 5 yielded the most energetically favourable result, with an estimated free binding energy of −9.47 kcal/mol and an inhibition constant (Ki) of 114.83 nM. Similar to the previous case, the analysis revealed the potential formation of five hydrogen bonds involving residues 121GLY, 203SER, 295PHE, 296ARG, and 447HIS. The image displays the spatial arrangement of the ligand within the protein structure (Figure 9C) and highlights the hydrogen bond with the highest probability (Figure 9D).

**Figure 10:**
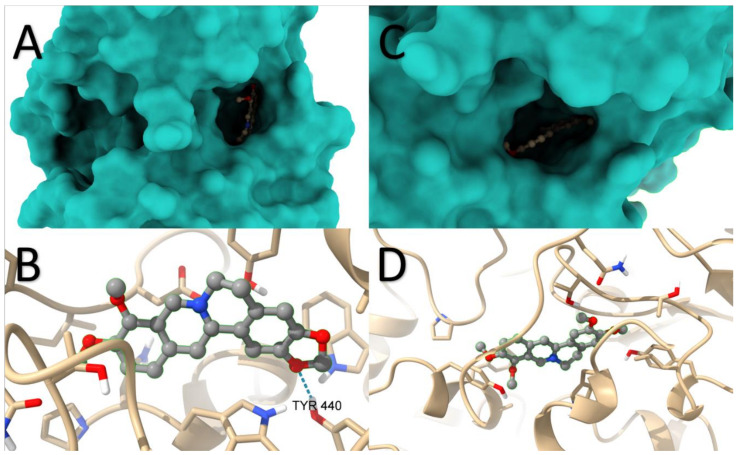
The location in BChE of berberine (**A**) and palmatine (**C**) along with the hydrogen bonds formed for berberine (**B**) and palmatine (**D**).

Docking studies with BChE and berberine produced 100 conformations, and RMSD cluster analysis was performed using all 25 ligand atoms. This analysis identified 18 distinct conformational clusters across the 100 runs, with a standard RMSD tolerance of 2.0 Å. The corresponding graph illustrates the distribution of runs among the various conformations, with run number 94 yielding the most favourable result. The estimated free binding energy was determined as −8.01 kcal/mol, derived from the final intermolecular energy (−8.61 kcal/mol), final total internal energy (−0.00 kcal/mol), torsional free energy (0.60 kcal/mol), and unbound system energy (−0.00 kcal/mol). The estimated inhibition constant (Ki) was calculated as 1340 nM (1.34 µM). Further analysis using the Protein-Ligand Interaction Profiler revealed the potential formation of four hydrogen bonds involving residues 70ASP, 82TRP, 430TRP, and 440TYR. The image displays the spatial arrangement of the ligand within the protein structure (Figure 10A) and highlights the hydrogen bond with the highest probability (Figure 10B).

Similarly, substituting berberine with palmatine resulted in 100 conformations. RMSD cluster analysis was performed using 26 ligand atoms, with a tolerance of 2.0 Å. The corresponding graph demonstrates the distribution of runs across the different conformations, totalling 34 in this case. Run number 37 yielded the most energetically favourable result, with an estimated free binding energy of −6.97 kcal/mol and an inhibition constant (Ki) of 7750 nM (7.75 µM). Consistent with the previous case, the analysis indicated the potential formation of two hydrogen bonds involving residues 120THR and 122THR. The image showcases the spatial arrangement of the ligand within the protein structure (Figure 10C) and highlights the hydrogen bond with the highest probability (Figure 10D).

Based on these results, it can be inferred that AChE is significantly more favourable for forming complexes with the proposed ligands compared to BChE The location in BChE of berberine and palmatine along with the hydrogen bonds formed for berberine and palmatine is presented in Figure 10. In the case of berberine, the estimated free binding energy with AChE was 116% higher than with BChE. For palmatine, this difference was even more pronounced, with a 136% advantage for AChE. Among the four investigated scenarios, the most stable complex was formed by palmatine with AChE, although it did not deviate significantly from the complex formed by berberine with AChE.

Furthermore, comparing these results with our previous work, where three other ligands, namely chelerythrine, protopine, and sanguinarine, were subjected to molecular docking analysis, provides valuable insights. The corresponding data is presented in the Table 6.

While previous studies demonstrated that BChE yielded considerably lower estimated free binding energy compared to AChE, the inclusion of berberine and palmatine as ligands resulted in a reversal of this trend and a significant improvement in the findings. Among all the collected data, the complexes formed by AChE with berberine and AChE with palmatine exhibit the most promising outcomes and warrant further investigation.

These findings indicate that AChE displays a higher affinity for berberine and palmatine compared to BChE, highlighting their potential as lead compounds for subsequent drug development. The enhanced binding affinities observed with AChE in the presence of berberine and palmatine make these combinations highly intriguing and deserving of additional research and exploration.

Lipophilicity is a very important property of drug candidate compounds. It is related to the pharmacokinetic parameters of biologically active molecules such as their absorption, distribution, metabolism, excretion, and toxicity. Some lipophilicity parameters (logP, the number of hydrogen bonds acceptor and donor, polar surface area) are presented in Table S1. More active alkaloids are characterized by higher log P values, while the least active protopine has the lowest log P value, a larger polar surface area compared to other alkaloids, and the highest number of hydrogen bond acceptor count. Lipophilicity parameters confirmed the ability of more active alkaloids to penetrate the blood–brain barrier.

### 2.7. In Vitro Determination of Cytotoxic Activity of Berberine and Plant Extracts

The cytotoxic activity of berberine and palmatine standards was investigated using the human melanoma cell line (A375). The MTT assay, which measures the activity of mitochondrial metabolism, was used for the determination of the influence of alkaloid standards on cancer cell viability. Berberine exhibited higher cytotoxic activity, with IC_50_ value of 51.6 μg/mL. The IC_50_ value obtained for human skin fibroblasts (WS1 cell line) was 95.316 μg/mL.

The same melanoma cell line in the same conditions as the previously investigated alkaloid standards was used for investigation of plant extract cytotoxicity (Table 7). The cytotoxic effect against A375 cells was observed especially at higher concentrations ranged from 25 to 200 μg/mL. Most investigated extracts showed moderate cytotoxic activity except the extract obtained from *Berberis gagnepainii,* which showed no cytotoxicity (IC_50_ > 200 μg/mL). For other extracts, IC_50_ values were in the range of 32.54–73.38 μg/mL. The highest cytotoxicity was observed for the extract obtained from the cortex of *Berberis pruinosa*. In order to evaluate the selectivity of the investigated extracts towards cancer cells, their cytotoxicity against human normal skin fibroblasts was also examined. The IC_50_ values obtained against human fibroblast cells (WS1) ranged from 58.36 μg/mL for *Berberis pruinosa* cortex extract to 94.32 μg/mL for extract obtained from the cortex of *Berberis aquifolium*. Different values of selectivity indexes were obtained for the investigated extracts; however, for the highest cytotoxic *Berberis pruinosa* extract, the selectivity index was 1.79 (similar to the selectivity index obtained for cisplatin), which indicates that the concentration of the extract for achieving therapeutic effects was lower than the concentration causing toxic effects.

Extracts obtained from various *Berberis* species were previously tested for cytotoxic activity against various other cancer cells. The cytotoxic activity of total alkaloids extracted from *Berberis hispanica* against human laryngeal carcinoma cells (Hep-2 cell line), with IC_50_ = 75 μg/mL, was reported [8]. In another study, the cytotoxic activity of *Berberis vulgaris* root extract showed cytotoxicity against breast (MCF7), liver (HepG2), and colon (CACO-2) cancer cell lines [10]. The viability of all tested cells was significantly decreased in a dose-dependent manner after treatment with both berberine chloride and *Berberis vulgaris* ethanolic extracts. The cytotoxic properties of the extract obtained from *Berberis aristata* root bark were tested against human rhabdomyosarcoma (RD), diploid mouse lung cell line (L20B), and human larynx epidermoid carcinoma (Hep 2) cell lines [11]. The cytotoxicity was observed against all tested cell lines with IC_50_ values ranged from 245 to 473 μg/mL.

### 2.8. Investigations of Toxicity of Berberis Pruinosa Extract on Danio rerio Larvae

The extract obtained from the cortex of *Berberis pruinosa,* showing the highest cytotoxic activity against A375 cells in in vitro experiments and an advantageous selectivity index value, was selected for in vivo experiments on *Danio rerio* larvae. The objective of the first step of in vivo studies was to estimate the maximum dose of the investigated extract that did not impair the development of *Danio rerio* embryos and larvae.

For determination of viability and deformity rate, embryos were exposed to E3 medium (control group) or serial dilutions of the *Berberis pruinosa* extract at concentrations of 1, 2.5, 5, 7.5, 10, 15, 25, and 50 μg/mL. 

The exposition of *Danio rerio* larvae to *Berberis pruinosa* extract at concentrations of 25 and 50 µg/mL caused a 100% mortality rate in the tested group of *Danio rerio* larvae after 96 hpf. Lower concentrations of the investigated extract did not cause a significant increase in the death rate (vs. the control group).

**Figure 11:**
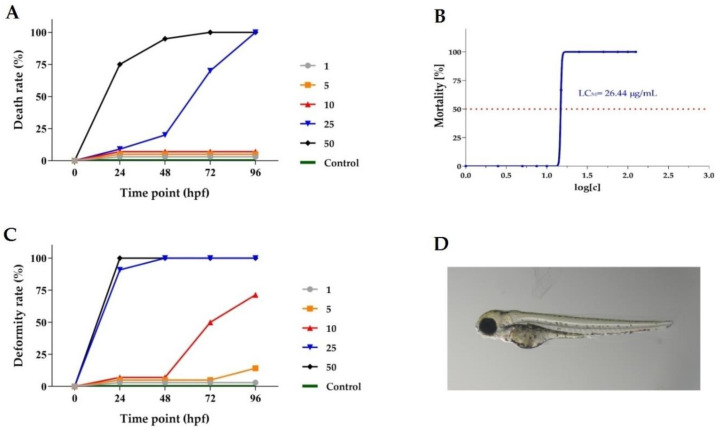
Analysis of *Berberis pruinosa* extract toxicity. (**A**) Dependence curves of *Danio rerio* embryo mortality rate on incubation time with a dilution series of *Berberis pruinosa* extract. (**B**) Dependence of *Danio rerio* larvae mortality on an exposition of various concentrations of *Berberis pruinosa* extract. The median lethal concentration (LC50) was calculated from cumulative mortality at 96 h postfertilization (hpf). (**C**) Time-response curves of deformity rate at tested doses. (**D**) Representative picture of 96 hpf zebrafish exposed to 10 μg/mL Berberis pruinosa extract.

The dependence of the mortality of *Danio rerio* larvae exposed to *Berberis pruinosa* extract at different concentrations on exposure time is presented in Figure 11A. Based on cumulative mortality obtained from three independent experiments for larvae at 96 h postfertilization, the median lethal concentration (LC_50_ = 26.44 μg/mL) was determined (Figure 11B). Figure 11C shows the dependence of deformities in *Danio rerio* larvae on the exposure time to various concentrations of the extract. Deformities were observed, especially after their exposition to the extract at concentrations of 25 and 50 μg/mL. After treatment with the investigated extract at concentrations of 5 μg/mL and lower after 72 h, no significant deformities were observed compared to the control group. In Figure 11D, a representative picture of *Danio rerio* larvae exposed at 96 hpf to 10 μg/mL *Berberis pruinosa* extract is shown.

### 2.9. Investigations In Vivo of Antitumour Activity of Berberis pruinosa Extract

**Figure 12:**
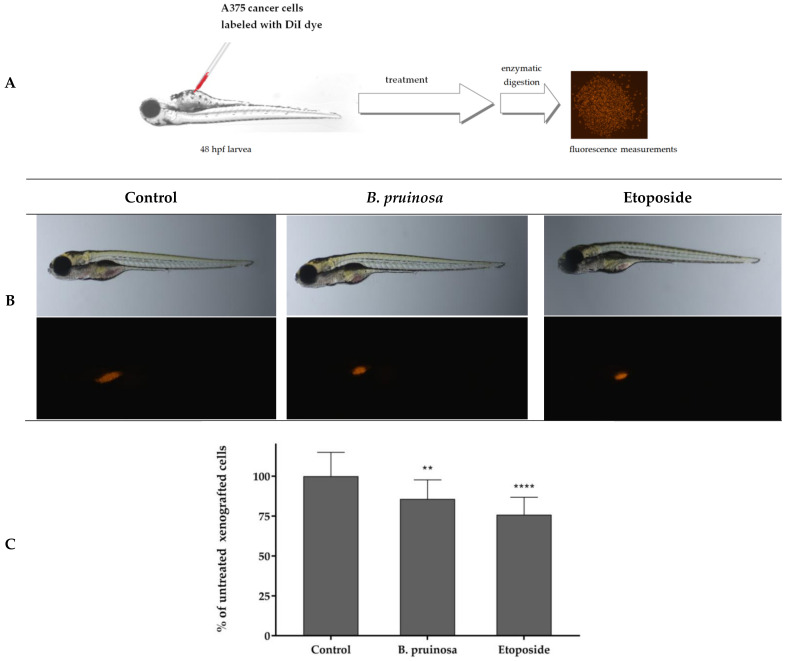
In vivo determination of *Berberis pruinosa* extract anticancer activity. (**A**) Scheme of the experiment based on *Danio rerio* human cancer cell xenograft model. (**B**) Images of 96 hpf (hours post fertilization) and 48 hpi (hours post injection) *Danio rerio* larvae xenografted with A375 cells treated with 5 μg/mL of *Berberis pruinosa* extract, 5 μg/mL of etoposide, or fish medium E3 (10 larvae/goup). (**C**) In vivo inhibition of cancer cell proliferation. ** *p* < 0.01 (vs. control group; one-way ANOVA), **** *p* < 0.0001.

In the next step, the human melanoma cancer cell line (A375) was xenografted into zebrafish embryos (an average of 500 cells). Xenografted zebrafish embryos were treated with 5 μg/mL of *Berberis pruinosa* extract, 5 μg/mL of etoposide, or fish medium E3. Cancer cells were injected into the center of the yolk sac. Figure 12A presents the scheme of the *Danio rerio* larvae xenograft experiments. The results obtained in our experiments showed a statistically significant moderate reduction, causing a reduction in tumour cell number in *Danio rerio* organisms after their exposition to the investigated extract (Figure 12B,C).

Currently gaining increasing attention of larval *Danio rerio* xenografts in investigations on new, effective candidates on anticancer drugs. *Danio rerio* has numerous advantages, such as easy maintenance, breeding, and transparent body during early development, ease administration of compound, short time and low cost of experiments, and especially, *Danio rerio* has a lot of physiological and genetic similarities with humans [22]. Larval *Danio rerio* xenograft model can be also applied for testing the response of cancer cells to different anti-cancer agents after a few days.

In vivo experiments using the *Danio rerio* xenograft model confirmed the high cytotoxic activity of the extract obtained from *Berberis pruinosa* cortex. The cytotoxic activity of *Berberis* species extracts against cancer cells has not been previously tested using in vivo experiments, while in vivo investigations have been performed for berberine against human tongue cancer SCC-4 cells using a murine xenograft model [23].

## 3. Experimental

### 3.1. HPLC-DAD Instrumentation and Conditions

A LC-20AD Shimadzu (Shimadzu Corporation, Canby, OR, USA) liquid chromatograph equipped with a Shimadzu SPD-M20A detector set in the 200–800 nm range (Shimadzu Corporation, Canby, OR, USA), thermostat CTO-10ASVP (Shimadzu Corporation, Canby, OR, USA), Rheodyne 20 μL injector, and column Synergi polar RP 80A (150 × 4.6 mm, 5 μm) (Phenomenex, Torrance, CA, USA) was used for analysis of alkaloid contents and cholinesterases activity. The eluent flow rate of 1.0 mL/min was used. The column oven temperature was maintained at 22 °C. A LabSolutions software version 5.71 (Shimadzu Corporation, Kyoto, Japan) was used for *the* acquisition and processing data. For the determination of alkaloid contents in plant extracts, previously described chromatographic system after appropriate modification was applied [16]. Solvent A was composed of 0.05 ML^−1^ 1-butyl-3-methylimidazolium tetrafluoroborate in water, and solvent B contained 1-butyl-3-methylimidazolium tetrafluoroborate in acetonitrile. The gradient program was as follows: 0–20 min, 30% B; 20–30 min, 30–55% B; 30–40 min, 55–60% B, 40–60 min, 60% B. 

### 3.2. LC-QqQ-ESI-MS/MS Parameters

Liquid chromatograph with the analytical column Kinetex C18 (100 mm × 2.1 mm, 1.7 µm) and a triple-quadrupole mass spectrometer (8050 Shimadzu (Kyoto, Japan)) equipped with LabSolution version 5.8 software for data collection and instrumental control were used. Electrospray ionization (ESI+) in the positive ion mode was applied. Before the analysis, the mass spectrometer was calibrated using the manufacturer’s calibration solution. The applied parameters were as follows: DL temperature 250 °C, interface temperature 300 °C, nebulizing gas flow 2 L/min, heat block temperature 400 °C, heating gas flow 10 L/min, drying gas flow 10 L/min, interface current 0.7 μA, interface voltage 4.0 kV, and temperature of drying gas 350 °C. Nitrogen was used as the collision gas, and the collision energy used was 25 eV. The mobile phase was composed of water (A) and methanol (B) with 0.1% formic acid, and the gradient program was as follows: 0–1 min, 25% B; 1–5 min, linear to 95% B; after that, returned to initial conditions at a flow rate of 0.4 mL/min and the temperature set at 30 °C.

### 3.3. Chemicals and Plant Materials

Acetonitrile (MeCN), methanol (MeOH), 1-butyl-3-methylimidazolium tetrafluoroborate, diethylamine, ammonium acetate and acetic acid of chromatographic quality were purchased from E. Merck (Darmstadt, Germany). acetylthiocholine iodide, butyrylthiocholine iodide, 5,5′-dithio-bis-(2-nitrobenzoic acid), TRIS hydrochloride, mercaptoethanol, acetylcholinesterase, and butyrylcholinesterase were purchased from Sigma-Aldrich (St. Louis, MO, USA).

The standard of palmatine was obtained from Chem Faces Biochemical Co., Ltd. (Wuhan, China), and the standard of berberine was obtained from Sigma-Aldrich (St. Louis, MO, USA). 

Plant material was collected in September 2021 and identified in the Botanical Garden of Maria Curie-Skłodowska University in Lublin (Poland). 

Fruits were dried at ambient temperature for 2 weeks. Branches of *Berberis* species were decorticated, and the cortex was dried for 2 weeks at ambient temperature. After grinding and mixing the plant material, 5 g of the raw material was weighed.

### 3.4. Extraction Procedure

The procedure of alkaloids extraction from plant material was based on a previously optimized procedure [24]. Plant material samples (5 g) were prepared by maceration with ethanol and extraction in an ultrasonic bath. After filtration and evaporation, the residues were dissolved in 2% sulfuric acid. The dissolved samples were defatted with diethylether. Next, after alkalization with 25% ammonia, alkaloids were extracted with chloroform. Organic solvent was evaporated, and the residue was dissolved in methanol.

### 3.5. Determination of Acetylcholinesterase Inhibitory Activity

The anti-acetylcholinesterase activity of alkaloid standards and *Berberis* species extracts was determined by the Ellman method with HPLC-DAD using the previously described procedure [25]. Analysis was performed on the same apparatus and conditions as analysis of alkaloid contents in plant extracts on Polar RP column using mobile phase consisted methanol and 0.0025 M diethylamine (A) and acetate buffer at pH 3.8 and 0.025 M diethylamine (B). The mobile phase’s gradient program was as follows: 0–10 min, 40% A; 10–30 min, 40–70% A; 30–60 min, 70% A. Samples were prepared by mixing acetylthiocholine iodide, 5′-dithiobis-(2-nitrobenzoic acid), acetylcholinesterase and phosphate buffer at pH 7.8. and incubated for 15 min at 37 °C. After filtration samples were injected into the HPLC system Final reaction product, 5-thio-2-nitro-benzoic acid was detected at λ = 405 nm. All experiments were repeated three times. Details are in Supplementary Materials (Section S1. Samples preparation for determination of acetylcholinesterase inhibitory activity).

### 3.6. Determination of Butyrylcholinesterase Inhibitory Activity

The same chromatographic conditions used for the determination of anti-acetylcholinesterase activity were applied for the determination of butyrylcholinesterase inhibitory activity. Samples were prepared by mixing butyrylthiocholine iodide, 5′-dithiobis-(2-nitrobenzoic acid), butyrylcholinesterase, and phosphate buffer at pH 7.8 and incubated at 37 °C for 15 min. Samples were filtered and injected into the HPLC system. 5-thio-2-nitro-benzoic acid was detected at λ = 405 nm. All experiments were performed in triplicates. For details, see Section S2. Samples preparation for determination of butyrylcholinesterase inhibitory activity.

### 3.7. Molecular Docking Methodology

Molecular docking is an essential tool for investigating and analysing interactions between receptor proteins and ligand molecules. Receptor structures for AChE (PDB ID: 4m0e) and BChE (PDB ID: 1p0p) were obtained in PDB format from the RCSB PDB website. The acquired structures were processed using Molegro Molecular Viewer 7 software to remove water molecules, attached ligands, and cofactors, ensuring a clean dataset. Ligand structures were obtained from the ZINC database. In contrast to protein structures, ligand structures were retrieved in mol2 format. Importantly, the consideration of Gasteiger charges was crucial for small-molecule ligands, as their charges are not preserved in the PDB format.

Molecular docking was performed using the blind mode of AutoDock Tools, an integral component of the MGL Tools suite. This mode facilitated an extensive exploration of the protein structure, allowing for the identification of potential binding sites for ligands. The protein structure remained rigid during the calculations, while the ligand exhibited conformational flexibility. The Lamarckian genetic algorithm version 4.2 was employed for the docking process. Subsequently, the obtained results and protein structures underwent comprehensive analysis using Microsoft Office and Chimera X software. Finally, the structures displaying the most promising outcomes, characterized by the highest negative energy and lowest inhibition, were visualized using the Protein-Ligand Interaction Profiler program.

### 3.8. Investigation of Cell Viability

Cytotoxicity of the Berberis species extracts, berberine and palmatine, was tested using a melanoma cell line (A375). For the evaluation of the effect of *Berberis* species, extracts against normal human skin fibroblast cells (WS1) were used. Both cell lines were received from the American Type Culture Collection (ATCC; Manassas, VA, USA). A375 and WS1 cells were cultured in Dulbecco’s Modified Eagle’s Medium (DMEM) (Sigma Aldrich, St. Louis, MO, USA). The procedure used in investigations of cell viability was described previously [26]. Inactivated fetal bovine serum, penicillin, and streptomycin were added to cells maintained in a CO_2_ atmosphere at 37 °C. The suspension of cells was applied to a 96-well plate and incubated for 24 h. Next, the medium was removed from the wells, and *Berberis* species extracts or alkaloid standards were added to the wells. After incubation, MTT working solution was added to each well, and the plate was again incubated for 3 h. Subsequently, an SDS solution was added, and the concentration of the dissolved formazan was measured (for details, see Section S3. Procedure for investigation of cell viability). For the calculation of IC_50_ values obtained for the plant extracts and alkaloid standards, the IC_50_ calculator (https://www.aatbio.com/tools/ic50-calculator, accessed on 16 January 2024) was used.

### 3.9. Danio rerio Culture and Fish Embryo Toxicity Test (FET)

The *Danio rerio* larvae xenograft experiment was performed according to the procedure described previously [26]. *Danio rerio* embryos were maintained in E3 embryo medium at 28 ± 0.5 °C. Embryos were exposed to the *Berberis pruinosa* extract at concentrations of 1, 2.5, 5, 7.5, 10, 15, 25, and 50 μg/mL or only E3 medium (control group). The effect of the final DMSO concentration on zebrafish development was not detectable. Further, 5 embryos per well, 10 per group, were placed in 24-well plates. The plates were maintained at 28 ± 0.5 °C under light/dark conditions (12 h/12 h). Viability and malformation rates of treatment embryos were determined at 24, 48, 72, and 96 hpf. Details of the procedure are described in Section S4. Procedure of *Danio rerio* culture and fish embryo toxicity test (FET). All experiments were performed in accordance with the National Institute of Health Guidelines for the Care and Use of Laboratory Animals and the European Community Council Directive for the Care and Use of Laboratory Animals (2010/63/EU). The agreement of the Local Ethical Commission for the experiments with larvae up to 5 dpf is not needed.

### 3.10. Danio rerio Human Tumour Cell Xenograft

*Danio rerio* embryos at 48 hpf were dechorionized and anesthetised, and A375 cancer cells were separated from culture dishes and washed with PBS.

Colouration of cells was performed with DiI diluted in PBS for 20 min at 37 °C. Cells were counted using microscopy, and injected into the centre of the yolk sac. Next, embryos were transferred into 96-well plates and incubated with *Berberis pruinosa* extract. After injection, embryos were maintained at 32 °C for 3 days, and cancer cell proliferation was analysed. The details of the procedure are described in Section S5. Procedure for Danio rerio human tumour cell xenograft.

### 3.11. Quantification of Xenografted Melanoma Cancer Cells

After 3 days post-injection (dpi), larvae were anaesthetized with tricaine and prepared the single-cell solution as described previously [27]. Next, cells were fixed with 50 μL of 8% paraformaldehyde solution. Images were made using a ZEISS SteREO Discovery.V8 microscope and Zen 2.3 lite software (Carl Zeiss Microscopy GmbH, Jena, Germany). The cells were counted using ImageJ software version 1.53 t.

### 3.12. Statistical Analysis

Statistical analysis was performed using GraphPad Prism 5.0 (GraphPad Software Inc., La Jolla, CA, USA).

## 4. Conclusions

Extracts obtained from various species of *Berberis* and its main alkaloids, berberine and palmatine, exhibited significant biological activities such as neuroprotection and cytotoxicity against cancer cells. 

Investigated isoquinoline alkaloids palmatine and especially berberine exhibited a cholinesterase inhibition activity. Berberine exhibited the activity, with IC_50_ = 2.04 μM against acetylcholinesterase and 12.42 μM against butyrylcholinesterase. 

Most investigated extracts also effectively inhibited cholinesterase activity. Extracts obtained from the cortex of *Berberis pruinosa* and *Berberis aquifolium* exhibited very high activity against acetylcholinesterase, with IC_50_ = 5.52 and 5.76 μg/mL, respectively. Obtained results showed that the extracts obtained from the cortex of *Berberis aquifolium* and *Berberis thunbergii* the highest inhibition of butyrylcholinesterase activity (IC_50_ = 10.45 and 26.12 μg/mL, respectively). Therefore, the extract obtained from the cortex of *Berberis aquifolium* shows to be the most promising in terms of the inhibition of both cholinesterases.

Based on the kinetic study, a non-competitive mechanism of acetylcholinesterase inhibition by berberine and a mixed mechanism by palmatine were found, while berberine inhibited butyrylcholinesterase activity in a mixed mode.

Most of the investigated plant extracts in the in vitro investigation also exhibited moderate but statistically significant cytotoxic activity against the human melanoma cell line (A375). The highest cytotoxicity was observed for extract obtained from the cortex of *Berberis pruinosa* with IC_50_ = 32.54 μg/mL. 

*Danio rerio* larvae xenograft model was used for the first time to determine *Berberis pruinosa* cortex extract cytotoxicity. The obtained results confirmed the in vivo antitumor activity of the investigated extract.

The extract obtained from the cortex of *Berberis gagnepainii* exhibited the lowest activity of both acetylcholinesterase inhibition and cytotoxic activity against the tested cancer cells; therefore, the extract is not recommended for further investigations in terms of these activities. Whereas, all other investigated extracts from the various *Berberis* species, especially *Berberis pruinosa,* can be recommended for further in vivo experiments. 

## Figures and Tables

**Table 1:** Equation of calibration curve, correlation coefficients (r), limit of detection (LOD), and limit of quantification (LOQ) values obtained for alkaloid standards.

Alkaloid	Equation of Calibration Curve	r	LOD [mg/mL]	LOQ [mg/mL]
Berberine	y = 91313877x − 1765457	0.9994	0.0204	0.0618
Palmatine	y = 76105127x + 1161612	0.9974	0.0513	0.1553

**Table 2:** Content of isoquinoline alkaloids in plant extracts and standard deviation of these values.

Species	Part of Plant	Berberine [mg/g of Dry Plant Material]	Palmatine [mg/g of Dry Plant Material]
*Berberis thunbergii*	Cortex	0.919 ± 0.082	0.055 ± 0.006
*Berberis thunbergii*	Fruits	0.212 ± 0.019	0.139 ± 0.009
*Berberis pruinosa*	Cortex	1.150 ± 0.101	0.181 ± 0.017
*Berberis gagnepainii*	Cortex	0.058 ±0.041	0.034 ± 0.004
*Berberis veitchii*	Cortex	0.156 ± 0.014	0.162 ± 0.015
*Berberis candidula*	Cortex	0.518 ± 0.052	0.358 ± 0.041
*Berberis aquifolium*	Cortex	0.166 ± 0.012	0.550 ± 0.058

**Table 3:** Factors and their levels with design matrix for the 2^3^ Central Composite Design (CCD).

Factor		Levels		
		Low (−1)	Central (0)	High (+1)
*LC-MS*				
	(X1) drying gas temperature [°C]	290	320	350
	(X2) nebulizer gas pressure [psi]	35	40	45
	(X3) capillary voltage [V]	3500	4000	4500
*Runs*		X1	X2	X3
	13	0	1.38	0
	6	1	−1	1
	2	−1	−1	1
	12	0	−1.7638	0
	10	−1.7638	0	0
	5	1	−1	−1
	9 (c)	0	0	0
	8	1	1	1
	4	−1	1	1
	11	1.38	0	0
	15	0	0	1.38
	3	−1	1	−1
	1	−1	−1	−1
	16 (c)	0	0	0
	7	1	1	−1
	14	0	0	−1.7638

**Table 4:** IC_50_ values obtained for alkaloid standards.

Alkaloid	IC_50_ Values			
	Acetylcholinesterase		Butyrylcholinesterase	
	μM	μg/mL	μM	μg/mL
Berberine	2.04 ± 0.07	0.69	12.42 ± 1.36	4.18
Palmatine	29.16 ± 1.59	10.27	189.40 ± 42.5	66.74
Galantamine	1.46 ± 0.02	0.42	6.31 ± 0.19	1.81
Rivastigmine	10.23 ± 0.88	2.56	8.38 ± 0.43	2.10

**Table 5:** IC_50_ values obtained for plant extracts.

Extract	IC_50_ Values (μg/mL)	
	Acetylcholinesterase	Butyrylcholinesterase
*Berberis thunbergii* cortex	11.02 ± 0.78	26.12 ± 1.14
*Berberis thunbergii* fruits	28.61 ± 1.46	>200
*Berberis pruinosa* cortex	5.52 ± 0.09	50.91 ± 11.3
*Berberis gagnepainii* cortex	>200	156.10
*Berberis veitchii* cortex	18.60 ± 1.57	40.40 ± 2.56
*Berberis candidula* cortex	18.46 ± 1.58	177.59
*Berberis aquifolium* cortex	5.76 ± 0.28	10.45 ± 0.47

**Table 6:** Comparison of data for selected proteins and ligands.

Receptor	Ligand	Estimated Free Energy of Binding (kcal/mol)	Estimated Inhibition Constant (Ki) (nM)	Hydrophobic Interactions	Hydrogen Bonds
AChE	Berberine	−9.30	152	5	5
	Palmatine	−9.47	115	7	5
	Chelerythrine	−7.50	3170	5	1
	Protopine	−6.51	16,990	3	1
	Sanguinarine	−7.50	3140	4	1
BCHE	Berberine	−8.01	1340	5	4
	Palmatine	−6.97	7750	3	2
	Chelerythrine	−8.18	1010	3	3
	Protopine	−8.36	750	1	3
	Sanguinarine	−8.14	1060	3	2

**Table 7:** Cytotoxic activity, expressed as IC_50_ values of the investigated extracts and the anticancer drug cisplatin against the melanoma cell line (A375) and human skin fibroblast cell line (WS1), and selectivity index.

Species	Part of Plant	IC_50_ [μg/mL]		Selectivity Index
		A375	WS1	
*Berberis thunbergii*	Cortex	51.6	64.24	1.24
*Berberis pruinosa*	Cortex	32.54	58.36	1.79
*Berberis gagnepainii*	Cortex	>200	>200	-
*Berberis veitchii*	Cortex	73.38	63.58	0.86
*Berberis candidula*	Cortex	61.83	60.14	0.97
*Berberis aquifolium*	Cortex	69.15	94.32	1.36
Cisplatin		23.8	44.07	1.85

## Data Availability

Data are contained within the article and Supplementary Materials.

## Supplementary Materials

The following supporting information can be downloaded at: https://www.mdpi.com/article/10.3390/molecules29051048/s1, Figure S1: Dependence of acetylcholinesterase activity on concentrations of alkaloid standards: berberine (1), palmatine (2), galantamine (3), and rivastigmine (4); Figure S2: Dependence of butylcholinesterase activity on concentrations of alkaloid standards: berberine (1), galantamine (2) and rivastigmine (3); Figure S3: Dependence of acetylcholinesterase activity on concentrations of plant extracts obtained from: *Berberis thunbergii* cortex (1), *Berberis thunbergii* fruits (2), *Berberis pruinosa* cortex (3), *Berberis veitchii* cortex (4), *Berberis candidula* cortex (5), *Berberis aquifolium* cortex (6); Figure S4: Dependence of acetylcholinesterase activity on concentrations of plant extracts obtained from: *Berberis thunbergii* cortex (1), *Berberis pruinosa* cortex (2), *Berberis aquifolium* cortex (3); Figure S5: Dependence of A375 human cancer cell viability on concentrations of plant extracts obtained from *Berberis thunbergii* cortex; Figure S6: Dependence of A375 human cancer cell viability on concentrations of plant extracts obtained from *Berberis pruinosa* cortex; Figure S7: Dependence of A375 human cancer cell viability on concentrations of plant extracts obtained from *Berberis veitchii* cortex; Figure S8: Dependence of A375 human cancer cell viability on concentrations of plant extracts obtained from *Berberis candidula* cortex; Figure S9: Dependence of A375 human cancer cell viability on concentrations of plant extracts obtained from *Berberis aquifolium* cortex; Figure S10: Dependence of WS1 human cancer cell viability on concentrations of plant extracts obtained from *Berberis thunbergii* cortex; Figure S11: Dependence of WS1 human cancer cell viability on concentrations of plant extracts obtained from *Berberis pruinosa* cortex; Figure S12: Dependence of WS1 human cancer cell viability on concentrations of plant extracts obtained from *Berberis veitchii* cortex; Figure S13: Dependence of WS1 human cancer cell viability on concentrations of plant extracts obtained from *Berberis candidula* cortex.; Figure S14: Dependence of WS1 human cancer cell viability on concentrations of plant extracts obtained from *Berberis aquifolium* cortex; Figure S15: Dependence of A375 human cancer cell viability on concentrations of cisplatin; Figure S16: Dependence of WS1 human cancer cell viability on concentrations of cisplatin; Table S1: Lipophilicity of alkaloid standards.

## References

[1] Plazas E., Avila M.M.C., Munoz D.R., Cuca S.L.E. (2022). Natural isoquinoline alkaloids: Pharmacological features and multi-target potential for complex diseases. Pharmacol. Res..

[2] Ahmed S., Khan S.T., Zargaham M.K., Khan A.U., Khan S., Hussain A., Uddin J., Khan A., Al-Harrasi A. (2021). Potential therapeutic natural products against Alzheimer’s disease with Reference of Acetylcholinesterase. Biomed. Pharmacother..

[3] Singh S., Pathak N., Fatima E., Negi A.S. (2021). Plant isoquinoline alkaloids: Advances in the chemistry and biology of berberine. Eur. J. Med. Chem..

[4] Malhotra B., Kulkarni G.T., Dhiman N., Joshi D.D., Chander S., Kharkwal A., Sharma A.K., Kharkwal H. (2021). Recent advances on *Berberis aristata* emphasizing berberine alkaloid including phytochemistry, pharmacology and drug delivery system. J. Herb. Med..

[5] Bonesi M., Loizzo M.R., Conforti F., Passalacqua N.G., Saab A., Menichinid F., Tundis R. (2013). *Berberis aetnensis* and *B. libanotica*: A comparative study on the chemical composition, inhibitory effect on key enzymes linked to Alzheimer’s disease and antioxidant activity. J. Pharm. Pharmacol..

[6] Kaufmann D., Dogra A.K., Tahrani A., Herrmann F., Wink M. (2016). Extracts from Traditional Chinese Medicinal Plants Inhibit Acetylcholinesterase, a Known Alzheimer’s Disease Target. Molecules.

[7] Hostalkova A., Marikova J., Opletal L., Korabecny J., Hulcova D., Kunes J., Novakova L., Perez D.I., Jun D., Kucera T. (2019). Isoquinoline Alkaloids from *Berberis vulgaris* as Potential Lead Compounds for the Treatment of Alzheimer’s Disease. J. Nat. Prod..

[8] Boudjlida A., Kaci S., Karaki S., Benayad T., Rocchi P., Smati D., Bouguerra Aouichat S. (2019). *Berberis hispanica* alkaloids extract induced cell death and apoptosis in human laryngeal cancer cells Hep-2. South Afr. J. Bot..

[9] Guo J., Li Y., Yu Z., Chen L., Chinnathambi A., Almoallim H.S., Alharbi S.A., Liu L. (2021). Novel green synthesis and characterization of a chemotherapeutic supplement by silver nanoparticles containing *Berberis thunbergii* leaf for the treatment of human pancreatic cancer. Biotechnol. Appl. Biochem..

[10] El-Wahab A.E.A., Ghareeb D.A., Sarhan E.E.M., Abu-Serie M.M., El Demellawy M.A. (2013). In vitro biological assessment of *berberis vulgaris* and its active constituent, berberine: Antioxidants, anti-acetylcholinesterase, anti-diabetic and anticancer effects. BMC Complemen. Altern. Med..

[11] Sood H., Kumar Y., Gupta V.K., Arora D.S. (2019). Scientific validation of the antimicrobial and antiproliferative potential of *Berberis aristata* DC root bark, its phytoconstituents and their biosafety. AMB Expr..

[12] Koutova D., Kulhava M., Havelek R., Majorosova M., Královec K., Habartova K., Hoštálková A., Opletal L., Cahlikova L., Rezácová M. (2020). Bersavine: A Novel Bisbenzylisoquinoline Alkaloid with Cytotoxic, Antiproliferative and Apoptosis-Inducing Effects on Human Leukemic Cells. Molecules.

[13] Rezadoost M.H., Kumleh H.H., Ghasempour A. (2019). Cytotoxicity and apoptosis induction in breast cancer, skin cancer and glioblastoma cells by plant extracts. Mol. Biol. Rep..

[14] Ahmad S., Hussain A., Hussain A., Abdullah I., Ali M.S., Froeyen M., Mirza M.U. (2019). Quantification of Berberine in *Berberis vulgaris* L. Root Extract and Its Curative and Prophylactic Role in Cisplatin-Induced In Vivo Toxicity and In Vitro Cytotoxicity. Antioxidants.

[15] Grissenberger S., Sturtzel C., Wenninger-Weinzierl A., Radic-Sarikas B., Scheuringer E., Bierbaumer L., Etienne V., Nemati F., Pascoal S., Totzl M. (2023). High-content drug screening in zebrafish xenografts reveals high efficacy of dual MCL-1/BCL-XL inhibition against Ewing sarcoma. Cancer Lett..

[16] Petruczynik A., Misiurek J., Tuzimski T., Waksmundzka-Hajnos M. (2017). Application of mobile phases containing ionic liquid for HPLC analysis of selected isoquinoline alkaloids. J. AOAC Int..

[17] Singh A., Bajpai V., Kumar S., Arya K.R., Sharma K.R., Kumar B. (2015). Quantitative determination of isoquinoline alkaloids and chlorogenic acid in *Berberis* species using ultra high performance liquid chromatography with hybrid triple quadrupole linear ion trap mass spectrometry. J. Sep. Sci..

[18] Belwal T., Pandey A., Bhatt I.D., Rawa R.S. (2020). Optimized microwave assisted extraction (MAe) of alkaloids and polyphenols from *Berberis* roots using multiple-component analysis. Sci. Rep..

[19] Tuzimski T., Petruczynik A., Szultka-Młynska M., Sugajski M., Buszewski B. (2022). Isoquinoline Alkaloid Contents in *Macleaya cordata* Extracts and Their Acetylcholinesterase and Butyrylcholinesterase Inhibition. Molecules.

[20] Fernández-Poyatos M., Ruiz-Medina A., Zengin G., Llorent-Martínez E.J. (2019). Phenolic Characterization, Antioxidant Activity and Enzyme Inhibitory Properties of *Berberis thunbergii* DC. Leaves: A Valuable Source of Phenolic Acids. Molecules.

[21] Mak S., Luk W.W.K., Cui W., Hu S., Tsim K.W.K., Han Y. (2014). Synergistic Inhibition on Acetylcholinesterase by the Combination Berberine and Palmatine Originally Isolated from Chinese Medicinal Herbs. J. Mol. Neurosci..

[22] MacRae C.A., Peterson R.T. (2015). Zebrafish as tools for drug discovery. Nat. Rev. Drug. Discov..

[23] Ho Y.-T., Yang J.-S., Lu C.-C., Chiang J.-H., Li T.-C., Lin J.-J., Lai K.-C., Liao C.-L., Lin J.-G., Chung J.-G. (2009). Berberine inhibits human tongue squamous carcinoma cancer tumor growth in a murine xenograft model. Phytomedicine.

[24] Petruczynik A., Misiurek J., Tuzimski T., Uszyński R., Szymczak G., Chernetskyy M., Waksmundzka-Hajnos M. (2016). Comparison of different HPLC systems for analysis of galantamine and lycorine in various species of *Amaryllidaceae* family. J. Liq. Chromatogr. Relat. Technol..

[25] Tuzimski T., Petruczynik A. (2021). Application of HPLC-DAD for In Vitro Investigation of Acetylcholinesterase Inhibition Activity of Selected Isoquinoline Alkaloids from *Sanguinaria canadensis* Extracts. Molecules.

[26] Tuzimski T., Petruczynik A., Plech T., Kaproń B., Makuch-Kocka A., Szultka-Młyńnska M., Misiurek J., Buszewski B., Waksmundzka-Hajnos M. (2023). Determination of Selected Isoquinoline Alkaloids from *Chelidonium majus*, *Mahonia aquifolium* and *Sanguinaria canadensis* Extracts by Liquid Chromatography and Their In Vitro and In Vivo Cytotoxic Activity against Human Cancer Cells. Int. J. Mol. Sci..

[27] Bresciani E., Broadbridge E., Liu P.P. (2018). An efficient dissociation protocol for generation of single cell suspension from zebrafish embryos and larvae. Methods X.

