# Supplementary material for: In Vitro and In Silico of Cholinesterases Inhibition and In Vitro and In Vivo Anti-Melanoma Activity Investigations of Extracts Obtained from Selected Berberis Species
Source: Molecules. 2024 Feb 28;29(5):1048. doi: 10.3390/molecules29051048 (PMC10933966; doi:10.3390/molecules29051048)
Supplement: Supplementary file 1 [file molecules-29-01048-s001.zip › molecules-2853445-supplementary.pdf]

S1. Samples preparation for determination of acetylcholinesterase inhibitory activity

A stock solutions of reagents were prepared by dissolving of acetylcholinesterase at concentrations 200 U/mL or 2 U/mL, acetylthiocholine iodide at concentration 0.0125 M and 5′-dithiobis-(2-nitrobenzoic acid) at concentration 0.00167 M in phosphate buffer at pH 7.8. Samples for determination of acetylcholinesterase inhibitory activity were obtained by mixing of 50 µL of acetylthiocholine iodide, 50 µL of 5′-dithiobis-(2-nitrobenzoic acid), 50 µL of acetylcholinesterase at concentration 2 U/mL and phosphate buffer at pH 7.8 to obtain 500 µL of final solution. In order to determine the IC50 value, alkaloid standards and extracts were added in the concentration range from 1 to 200 ug/ml. The volume of added buffer was reduced by an appropriate volume so that the final concentrations of the remaining reagents were constant. Concentration of acetylcholinesterase in samples for determination of IC50 values was 0.2 U/mL. After mixing of all components samples were incubated for 15 min at 37°C. Before injected into HPLC system samples were filtered by syringe filter (CHROMAFIL Xtra, PVDF-29/25 0.20 µm). Final reaction product, 5-thio-2-nitro-benzoic acid was detected at λ=405 nm. All experiments were repeated three times.

S2. Samples preparation for determination of butyrylcholinesterase inhibitory activity

A stock solutions were prepared by dissolving of butyrylcholinesterase at concentration of 200 U/mL and 2 U/mL, butyrylthiocholine iodide at concentration 0.0125 M and 5′-dithiobis-(2-nitrobenzoic acid) at concentration 0.00167 M in phosphate buffer at pH 7.8. For determination of butylcholinesterase inhibitory activity reaction mixtures were prepared by mixing of 50 µL of acetylthiocholine iodide, 50 µL of 5′-dithiobis-(2-nitrobenzoic acid), 50 µL of butyrylcholinesterase at concentration 2 U/mL and phosphate buffer at pH 7.8 to obtain 500 µL of final solution. In order to determine the IC50 value, alkaloid standards and extracts were added in the concentration range from 1 to 200 ug/ml. The volume of added buffer was reduced by an appropriate volume so that the final concentrations of the remaining reagents were constant. For determination of IC50 values final concentration of butyrylcholinesterase was 0.2 U/mL. Samples were incubat-ed at 37°C for 15 min. Samples were filtered by syringe filter (CHROMAFIL Xtra, PVDF-29/25 0.20 µm) before injection into HPLC system. 5-thio-2-nitro-benzoic acid was detected at λ=405 nm. All experiments were performed in triplicates.

S3. Procedure for investigation of cell viability

100 U/mL of penicillin, and 100 µg/mL streptomycin. The cells were maintained in a 5% CO2 atmosphere at 37 °C. Stock solutions at concentrations 50 mg/mL of palmatine, ber-berine and dried Berberis species extracts were prepared by dissolving appropriate amount of them in DMSO. At the day of the experiment, the suspension of cells (1 × 105 cells/mL) in the respective medium was applied to a 96-wellplate at 100 µL per well. Cells were incubated after 24h, subsequently the medium was removed from the wells. Berberis species extracts or alkaloid standards in medium containing 2% FBS atiIncreasing con-centrations were added the wells. The human skin fibroblasts were only cultured with a medium containing 2% FBS. Cytotoxicity of DMSO was also determined at concentrations present in respective dilutions of stock solutions. DMSO in the used concentrations did not influenced the viability of the investigated cells. After 24 h of incubation, 15 µL MTT working solution (5 mg/mL in PBS)

was added to each well. Then, plate were incubated for 3 h. After incubation 100 µL of 10% SDS solution was added to each well. Subsequent-ly, using a microplate reader (Epoch, BioTek Instruments, Inc., Winooski, VT, USA) deter-mination of concentration of the dissolved formazan was performed by measuring the absorbance at λ = 570 nm. Three replicates were performed for two independent experi-ments. DMSO at the concentrations used did not affect the viability of the tested cells.

S4. Procedure of Danio rerio culture and fish embryo toxicity test (FET)

Danio rerio of the AB strain (Experimental Medicine Centre, Medical University of Lu-blin, Poland) were maintained under a 14/10 h light/dark cycle at 28 ± 0.5°C, with stand-ard aquaculture conditions. The fertilized eggs were collected within 30 min after mating. Embryos were reared in E3 embryo medium (pH 7.1–7.3) containing 17.4 µM NaCl, 0.21 µM KCl, 0.12 µM MgSO4 and 0.18 µM Ca(NO3)2) in an incubator (IN 110 Memmert GmbH, Germany) at 28 ± 0.5°C. Freshly prepared stock solution of plant extract was di-luted in E3 embryo medium in order to receive the appropriate treatment concentrations. Embryos were exposed to the Berberis pruinosa extract at concentrations of 1, 2.5, 5, 7.5, 10, 15, 25, 50 µg/mL or only E3 medium (control group). The effect of the final DMSO con-centration on zebrafish development was not detectable. 5 embryos per well, 10 per group were placed in 24-well plates. All experiments were performed in triplicates. Next, the covered plates were kept at 28 ± 0.5°C under the light/dark conditions (12h/12h). Viability, and malformation rates of each treatment group of embryos were recorded at 24, 48, 72, and 96 hpf.

S5. Procedure for Danio rerio human tumor cell xenograft

Danio rerio embryos at 48 hpf were dechorionized using microforceps, anesthetized with 0.0016% tricaine, and positioned on their left side on a wet Petri dish with micro-scope slide. A375 cancer cells were separated from culture dishes using 0.25% Tryp-sin-EDTA and washed twice with PBS at room temperature.

Coloration of cells were performed with 5 µM DiI diluted in PBS, for 20 minutes at 37°C. Next, A375 cells were washed three times with PBS. Cells were counted using mi-croscopy, and injected into the center of the yolk sac by an microinjector (NARISHIGE, IM-300, Japan) with micromanipulator (World Precision Instruments, 3301R, USA) equipped with borosilicate glass capillaries (World Precision Instruments, Sarasota, USA). Next, embryos were transfered into 96-well plates and incubated with Berberis pruinosa extract diluted in E3 media at concentration of 5 µg/mL. The control group consisted of injected embryos in E3 medium. After injection embryos were maintained at 32°C for 3 days and cancer cell proliferation was analyzed.

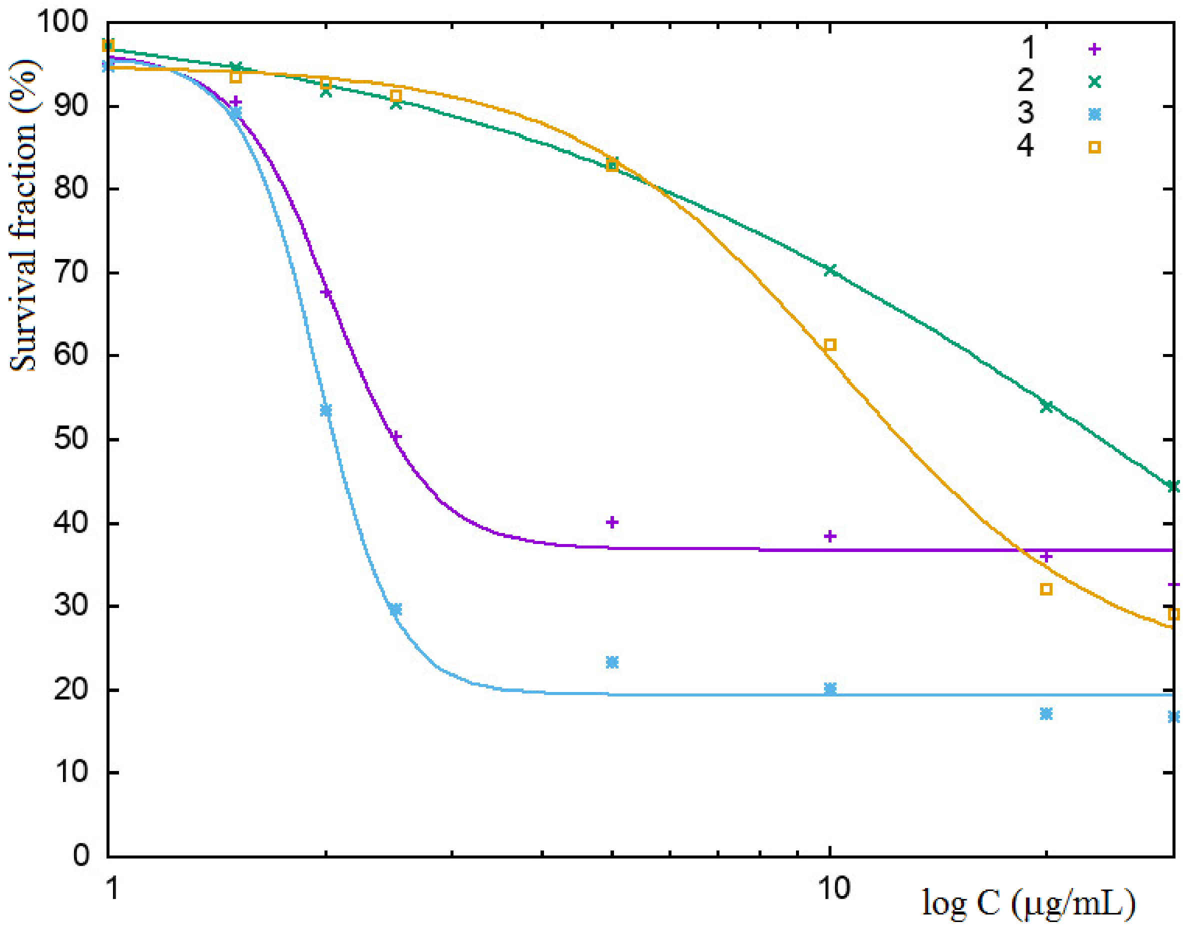

Figure S1. Dependence of acetylcholinesterase activity on concentrations of alkaloid standards: berberine (1), palmatine (2), galantamine (3) and rivastigmine (4).

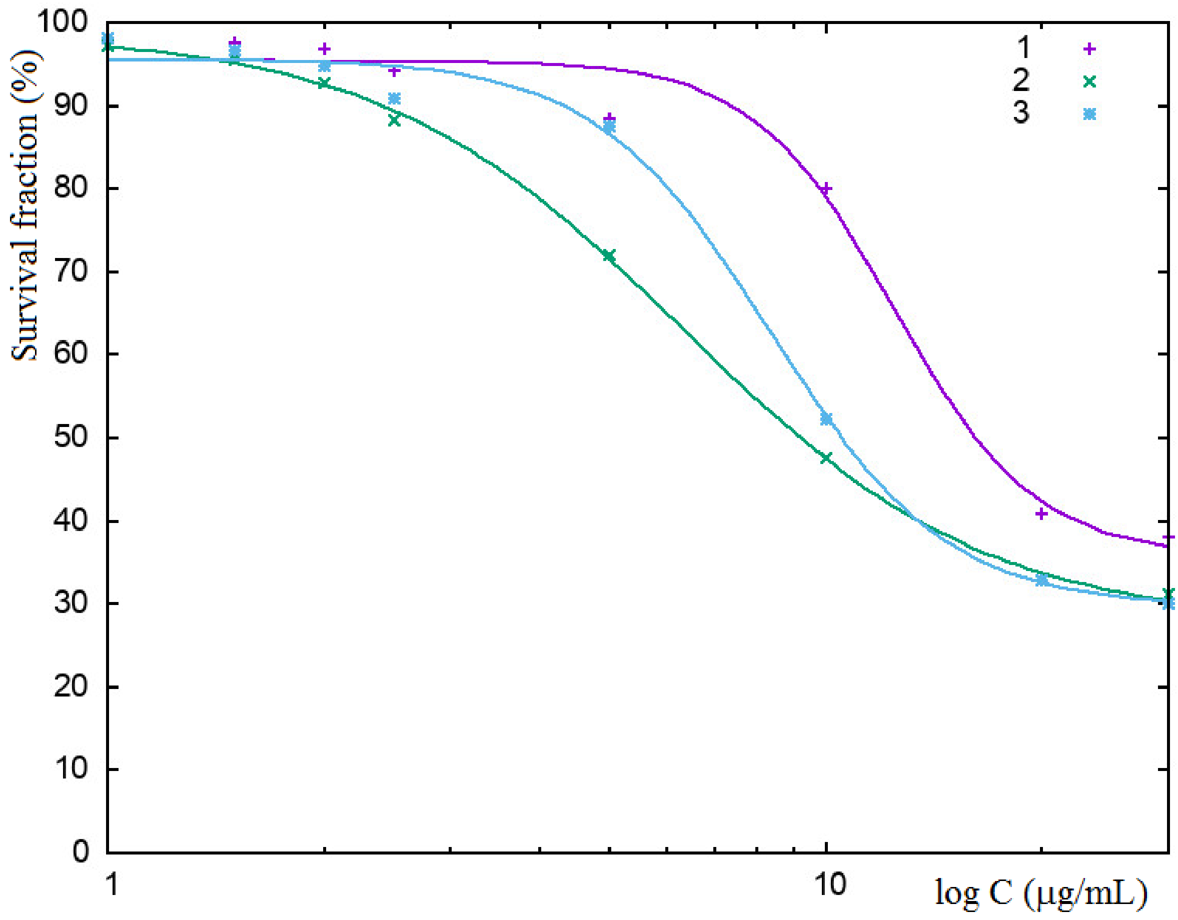

Figure S2. Dependence of butylcholinesterase activity on concentrations of alkaloid standards: berberine (1),), galantamine (2) and rivastigmine (3).

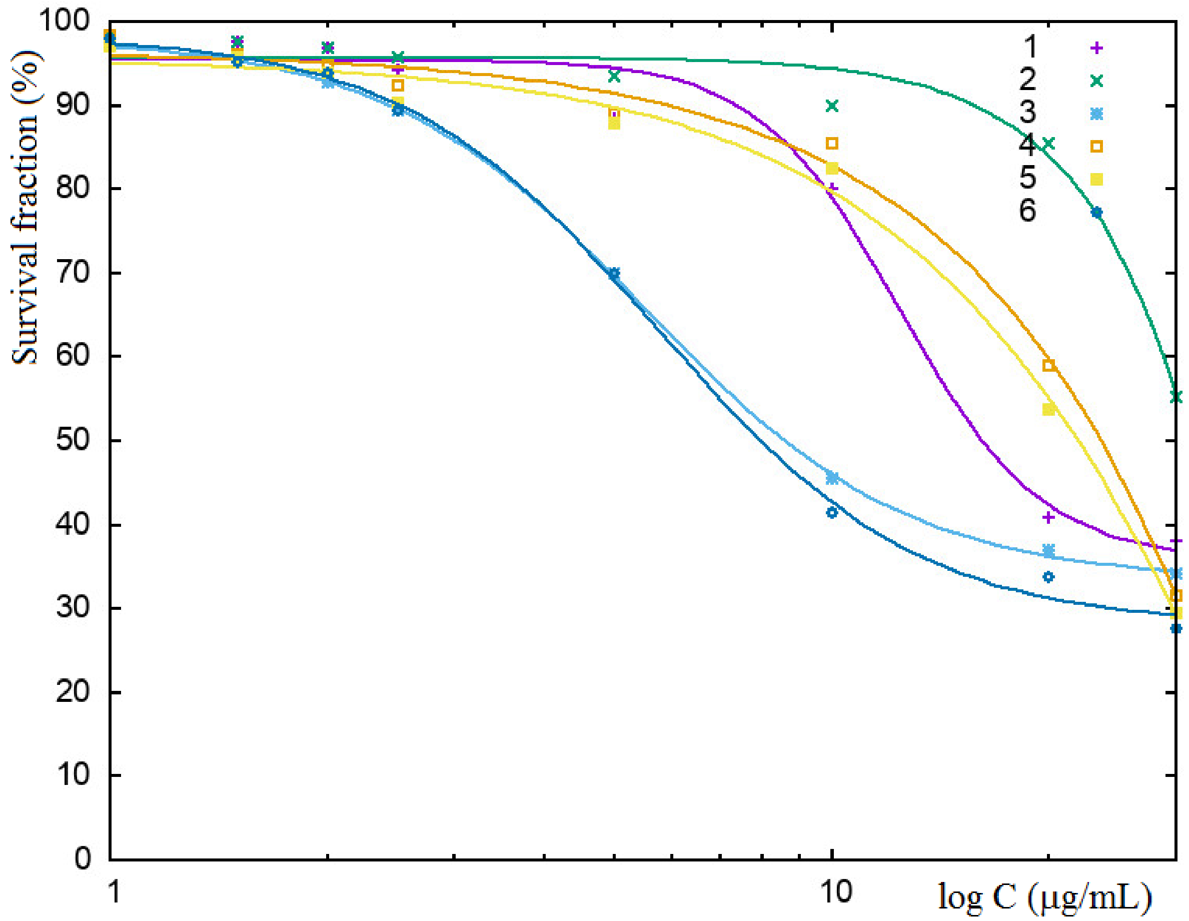

Figure S3. Dependence of acetylcholinesterase activity on concentrations of plant extracts obtained from: *Berberis thunbergii* cortex (1), *Berberis thunbergii* fruits (2), *Berberis pruinosa* cortex (3), *Berberis veitchii* cortex (4), *Berberis candidula* cortex (5), *Berberis aquifolium* cortex (6).

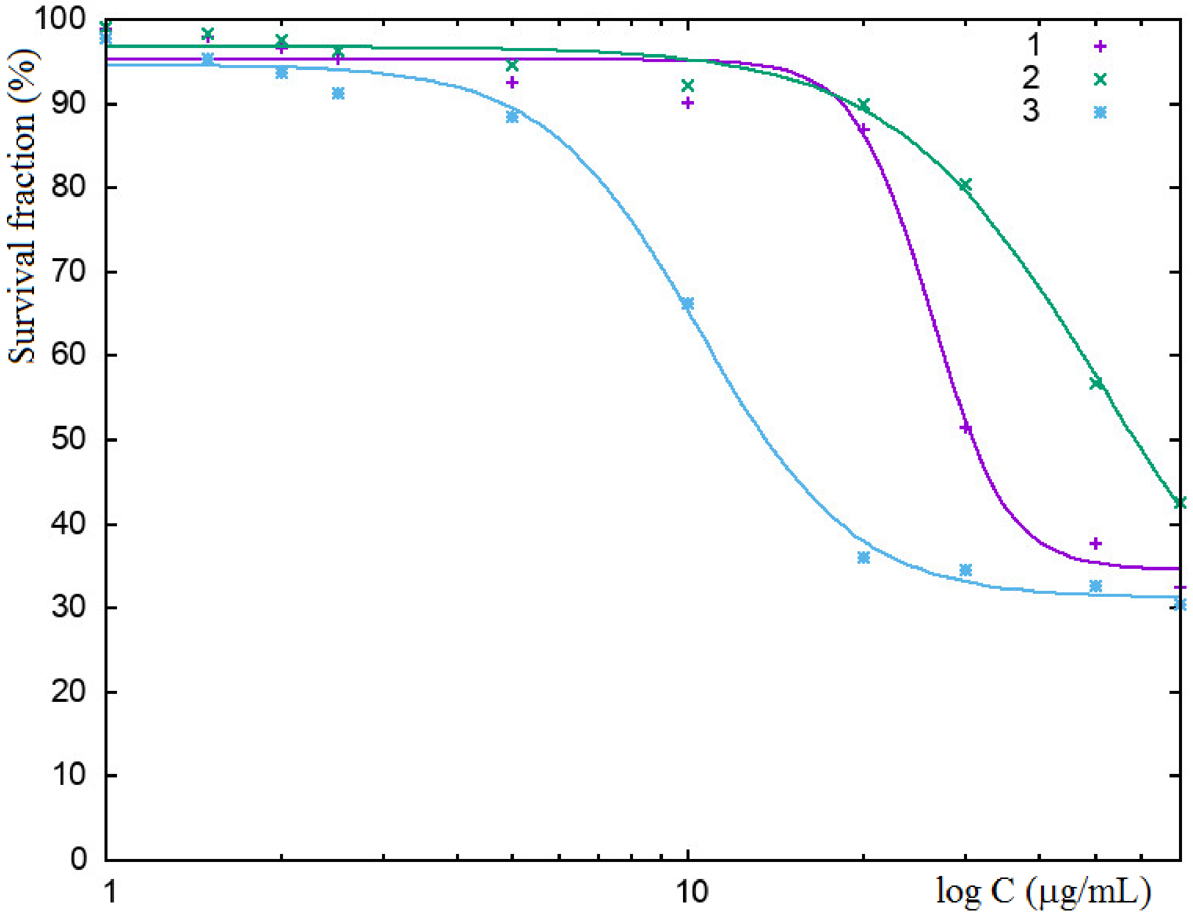

Figure S4. Dependence of acetylcholinesterase activity on concentrations of plant extracts obtained from: *Berberis thunbergii* cortex (1), *Berberis pruinosa* cortex (2), *Berberis aquifolium* cortex (3).

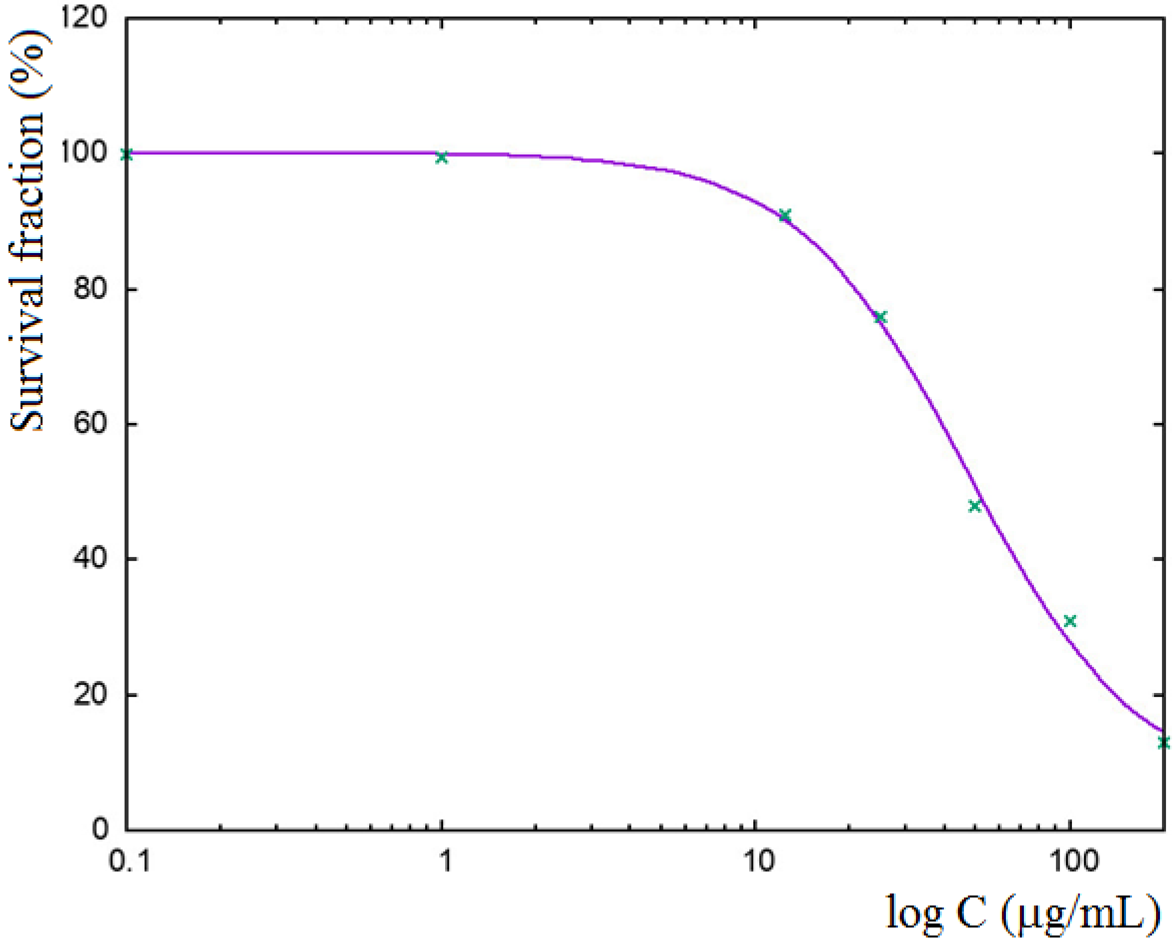

Figure S5. Dependence of A375 human cancer cell viability on concentrations of plant extracts obtained from *Berberis thunbergii* cortex.

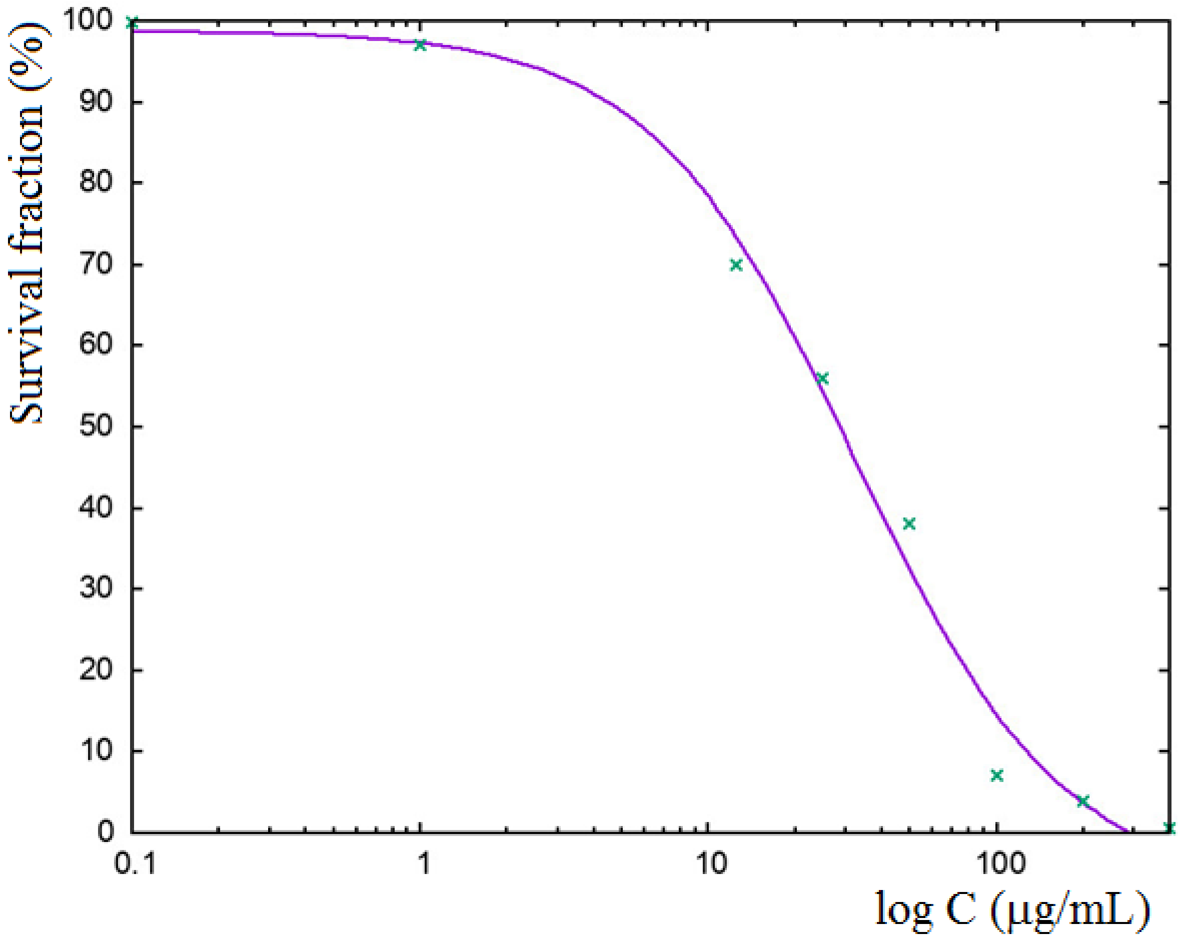

Figure S6. Dependence of A375 human cancer cell viability on concentrations of plant extracts obtained from *Berberis pruinosa* cortex.

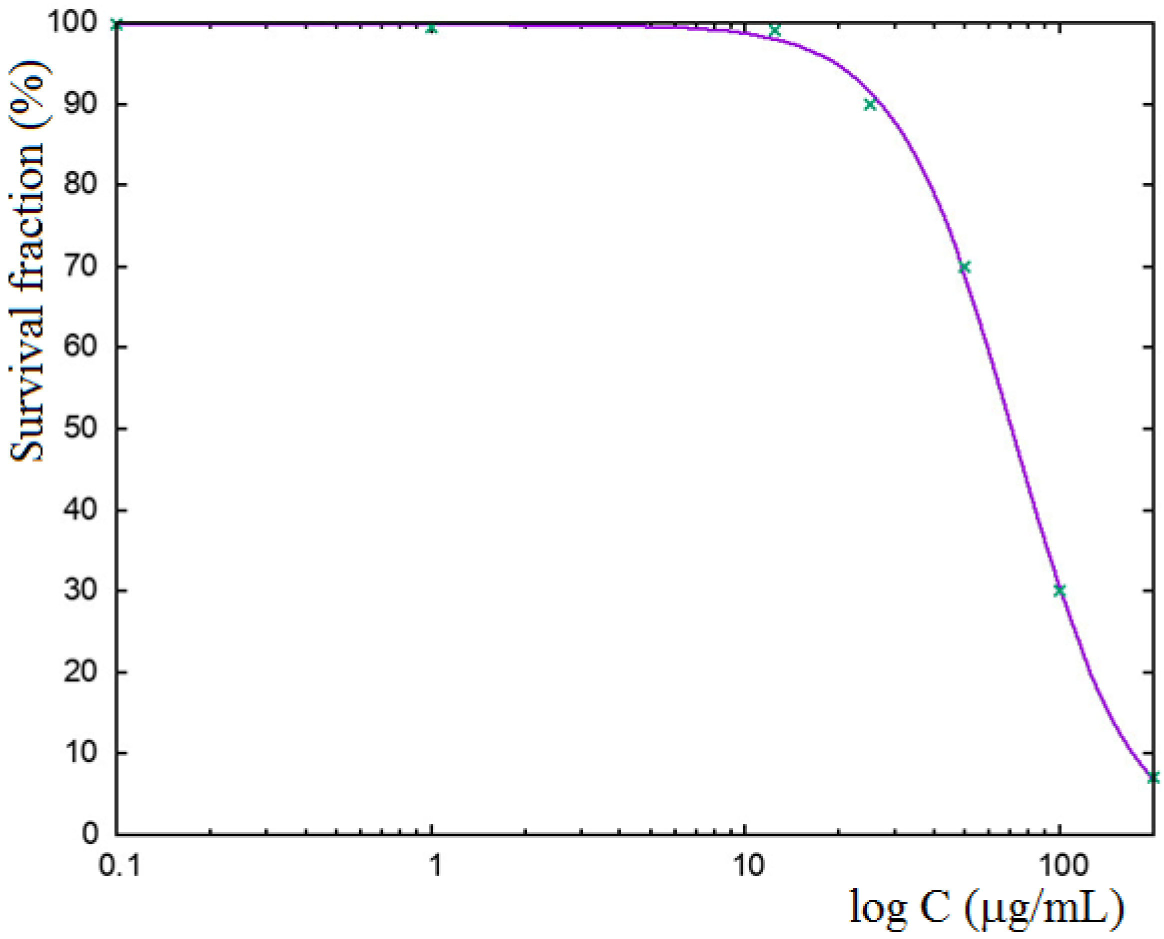

Figure S7. Dependence of A375 human cancer cell viability on concentrations of plant extracts obtained from *Berberis veitchii* cortex.

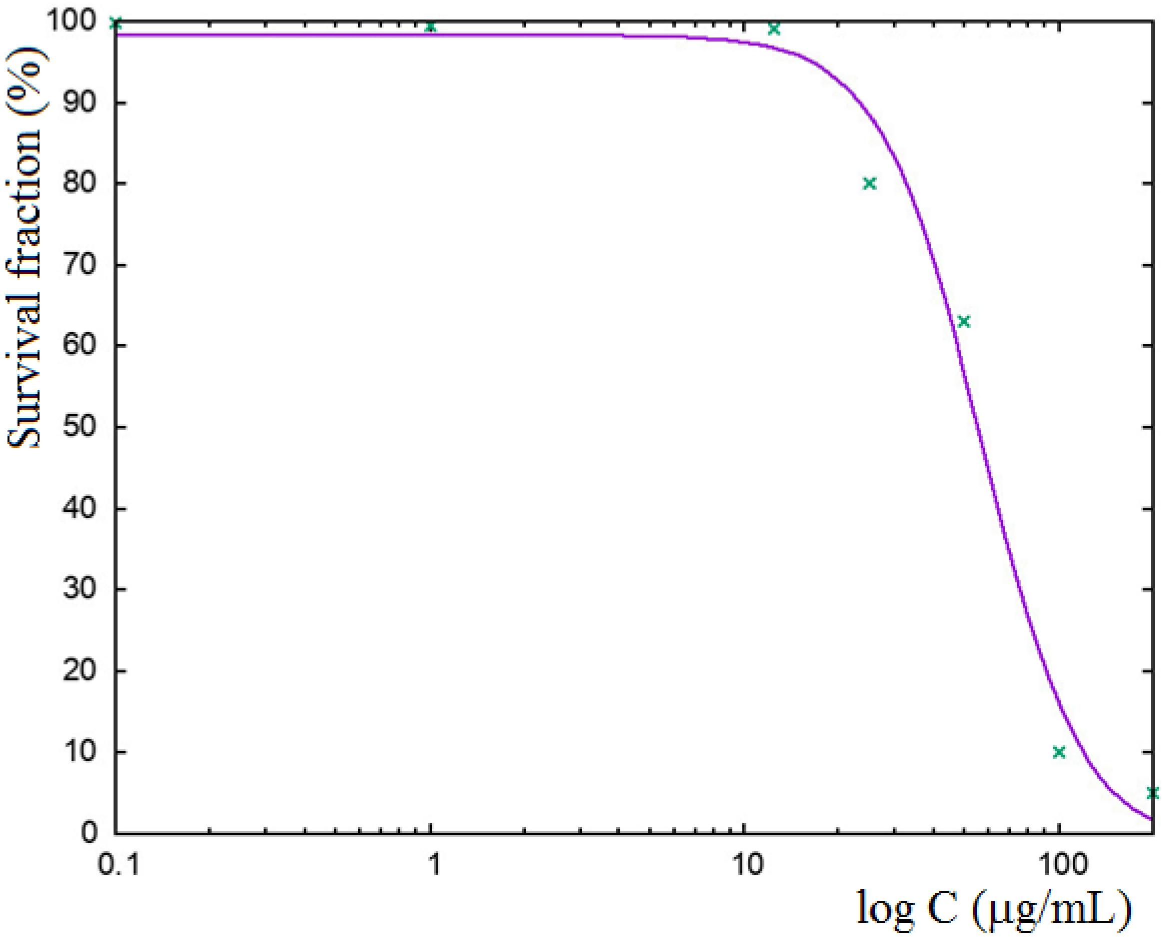

Figure S8. Dependence of A375 human cancer cell viability on concentrations of plant extracts obtained from *Berberis candidula* cortex.

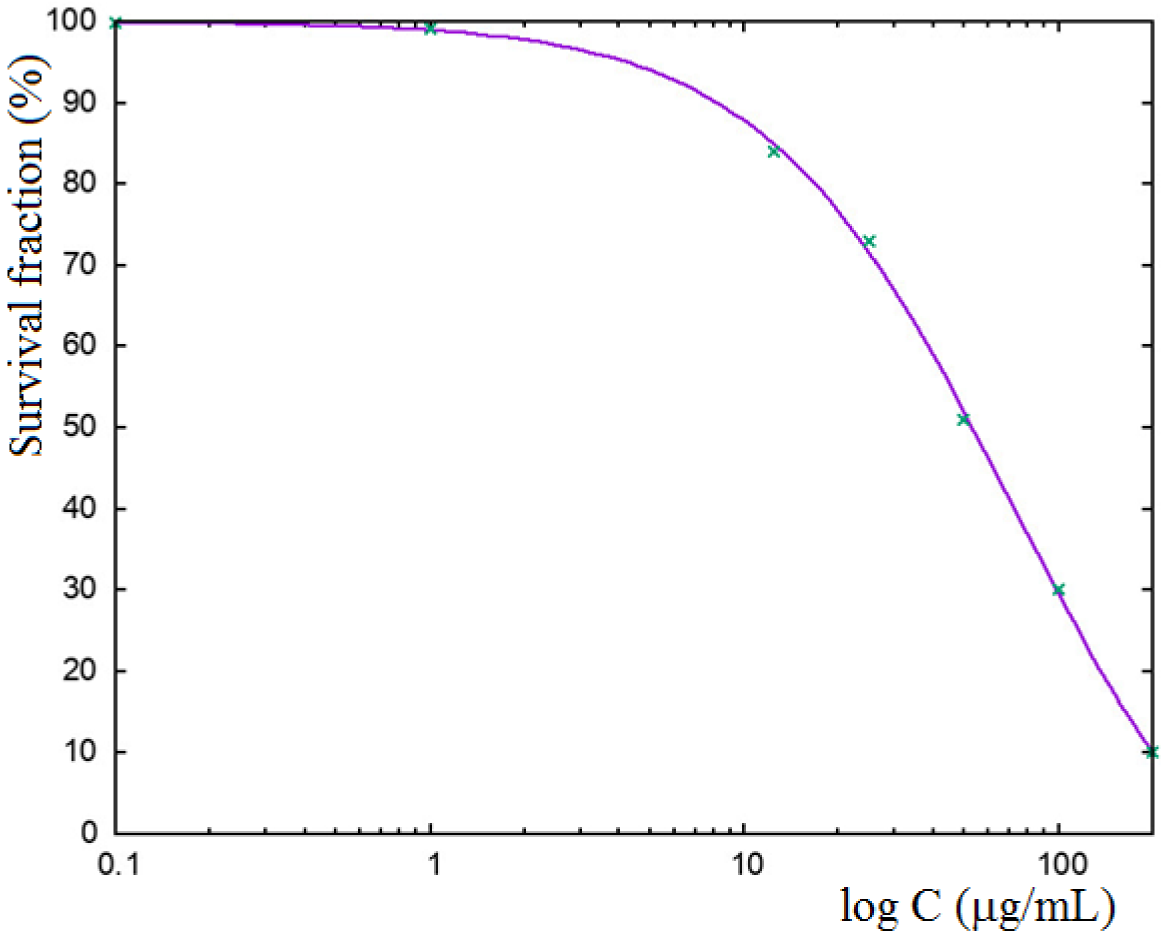

Figure S9. Dependence of A375 human cancer cell viability on concentrations of plant extracts obtained from *Berberis aquifolium* cortex.

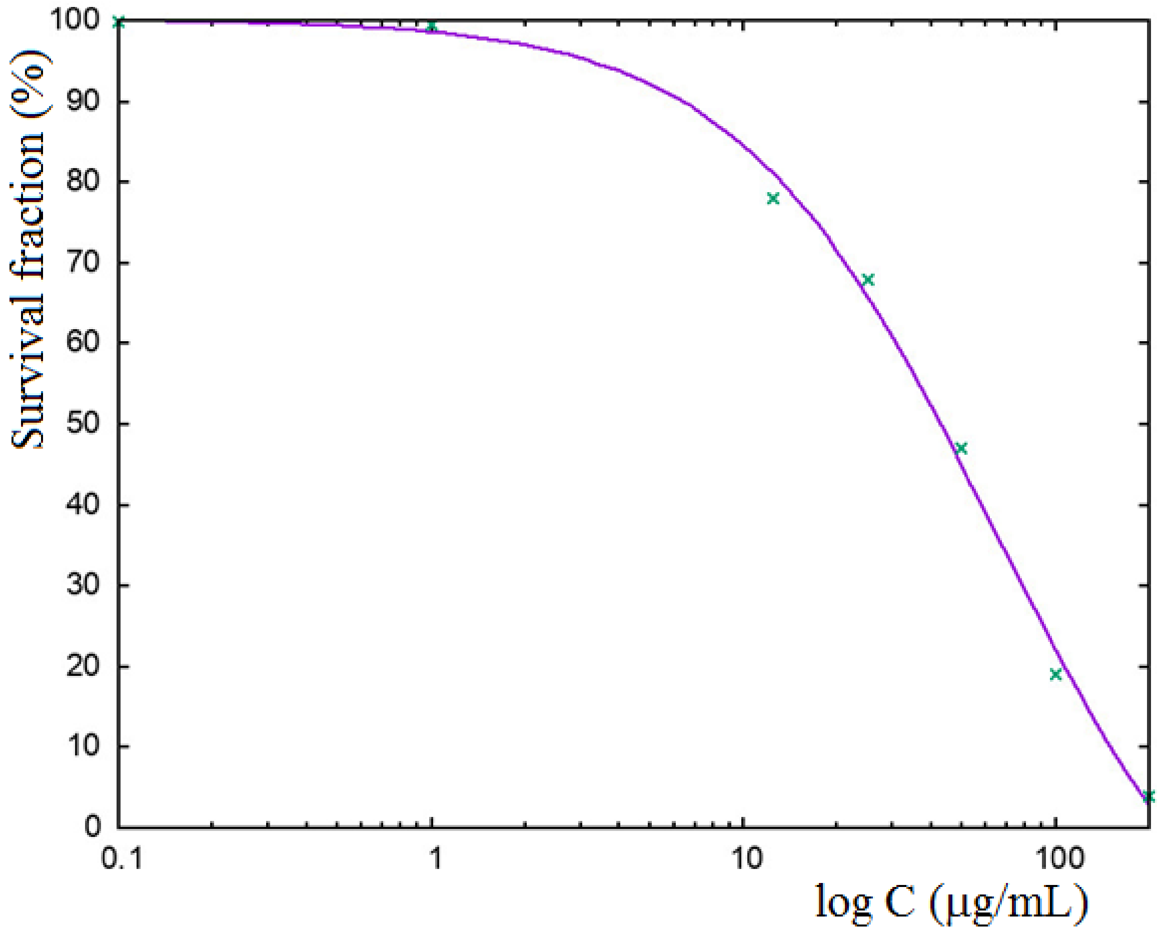

Figure S10. Dependence of WS1 human cancer cell viability on concentrations of plant extracts obtained from *Berberis thunbergii* cortex.

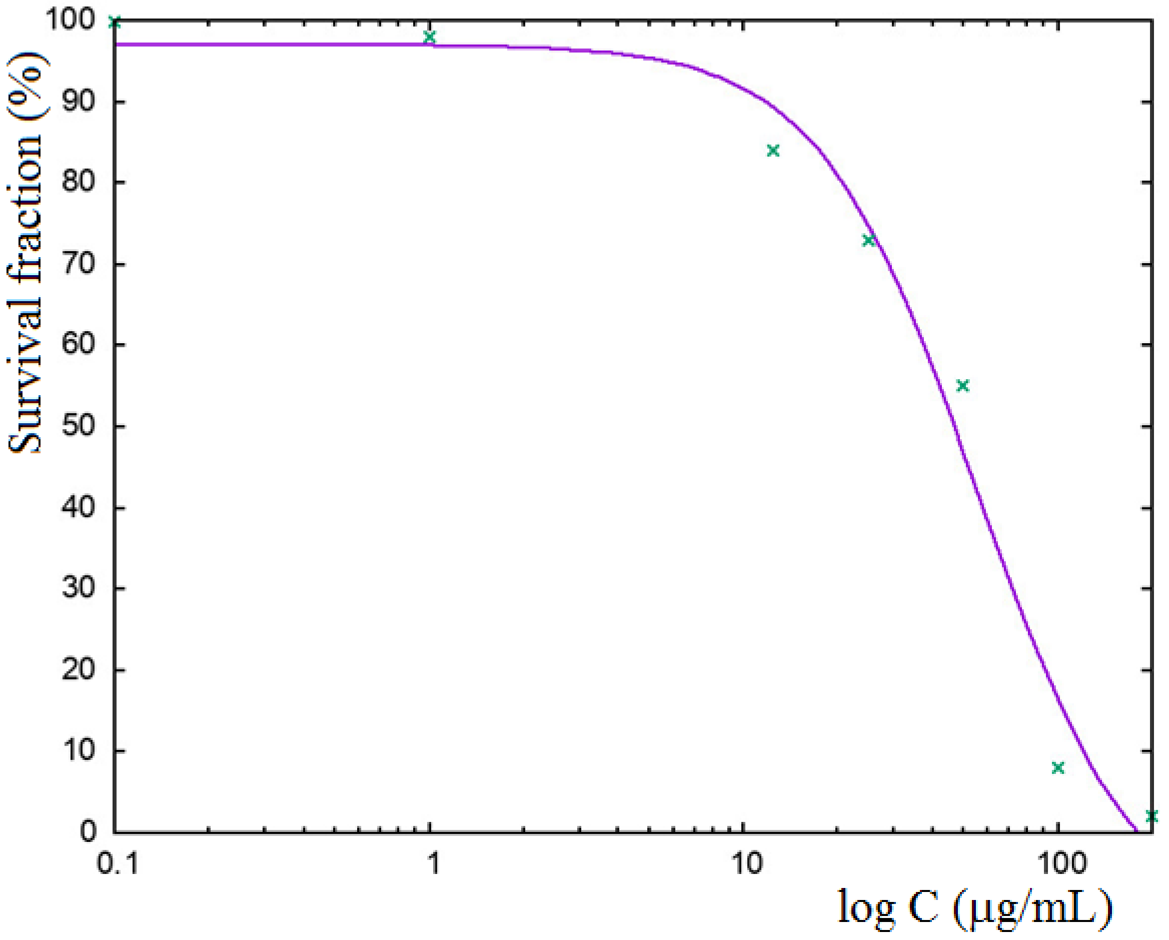

Figure S11. Dependence of WS1 human cancer cell viability on concentrations of plant extracts obtained from *Berberis pruinosa* cortex.

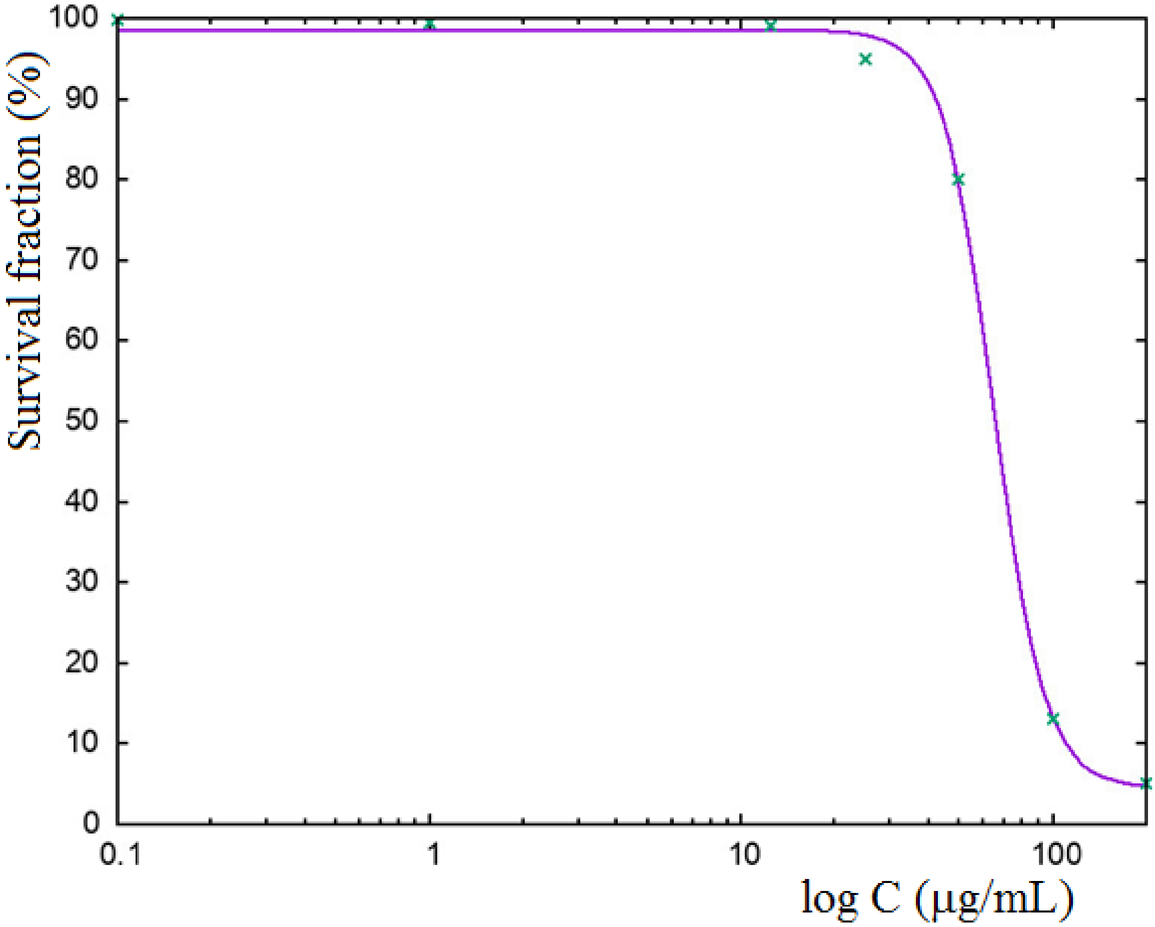

Figure S12. Dependence of WS1 human cancer cell viability on concentrations of plant extracts obtained from *Berberis veitchii* cortex.

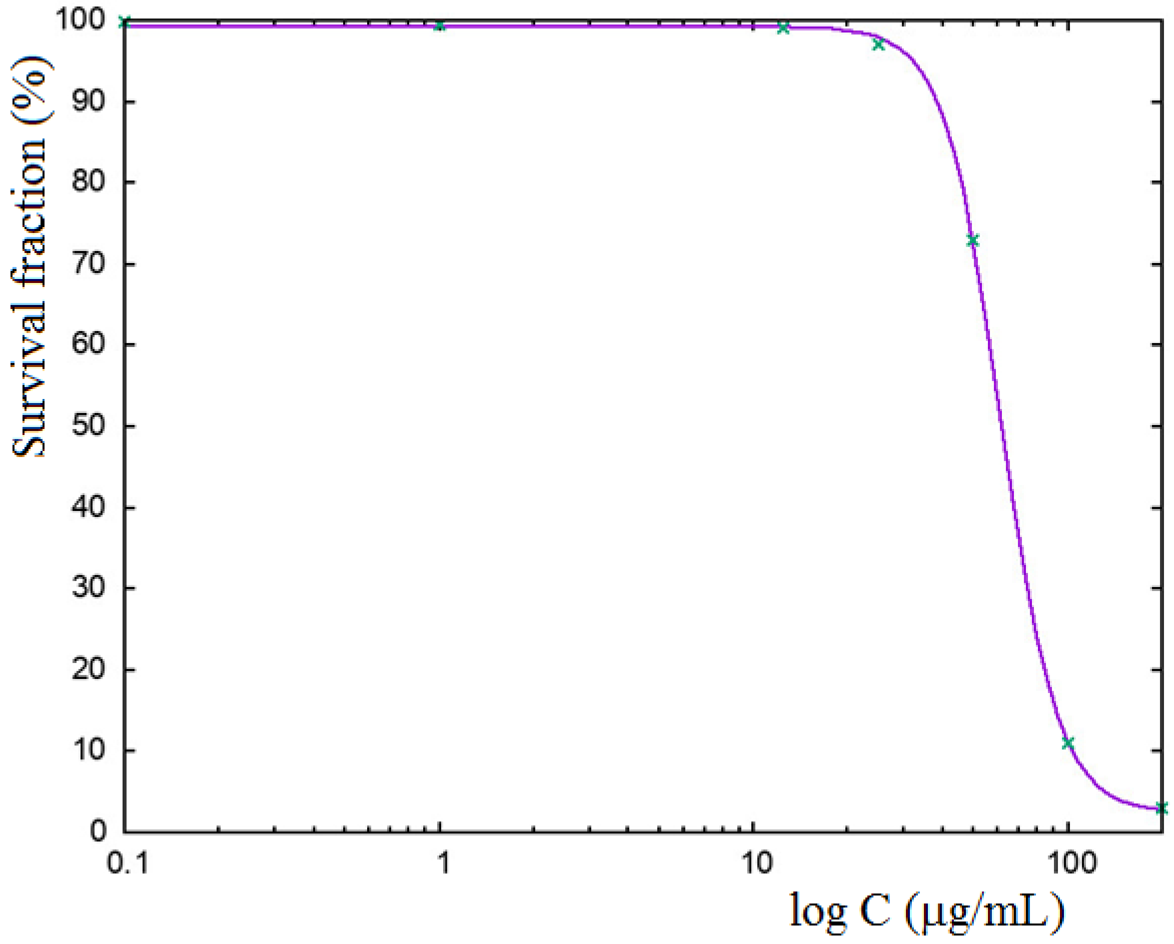

Figure S13. Dependence of WS1 human cancer cell viability on concentrations of plant extracts obtained from *Berberis candidula* cortex.

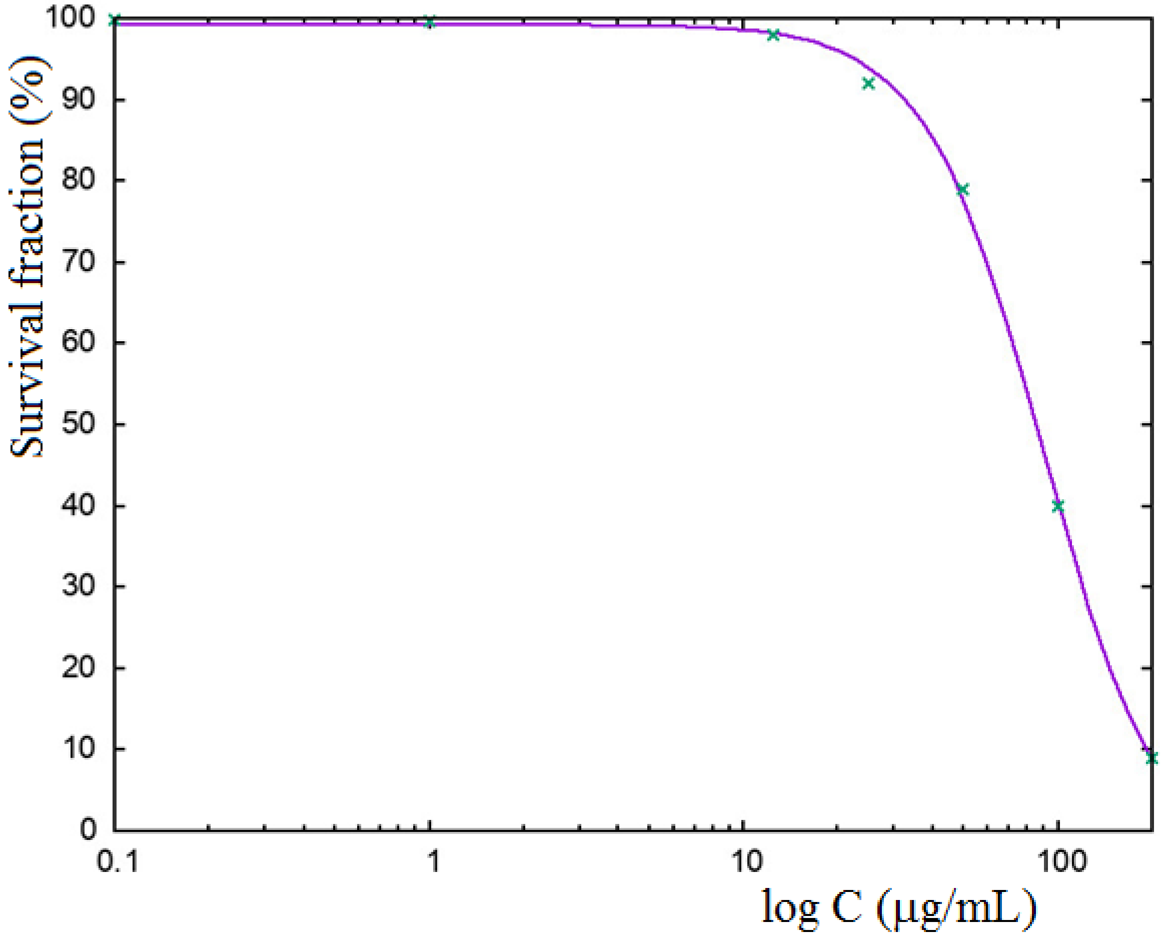

Figure S14. Dependence of WS1 human cancer cell viability on concentrations of plant extracts obtained from *Berberis aquifolium* cortex.

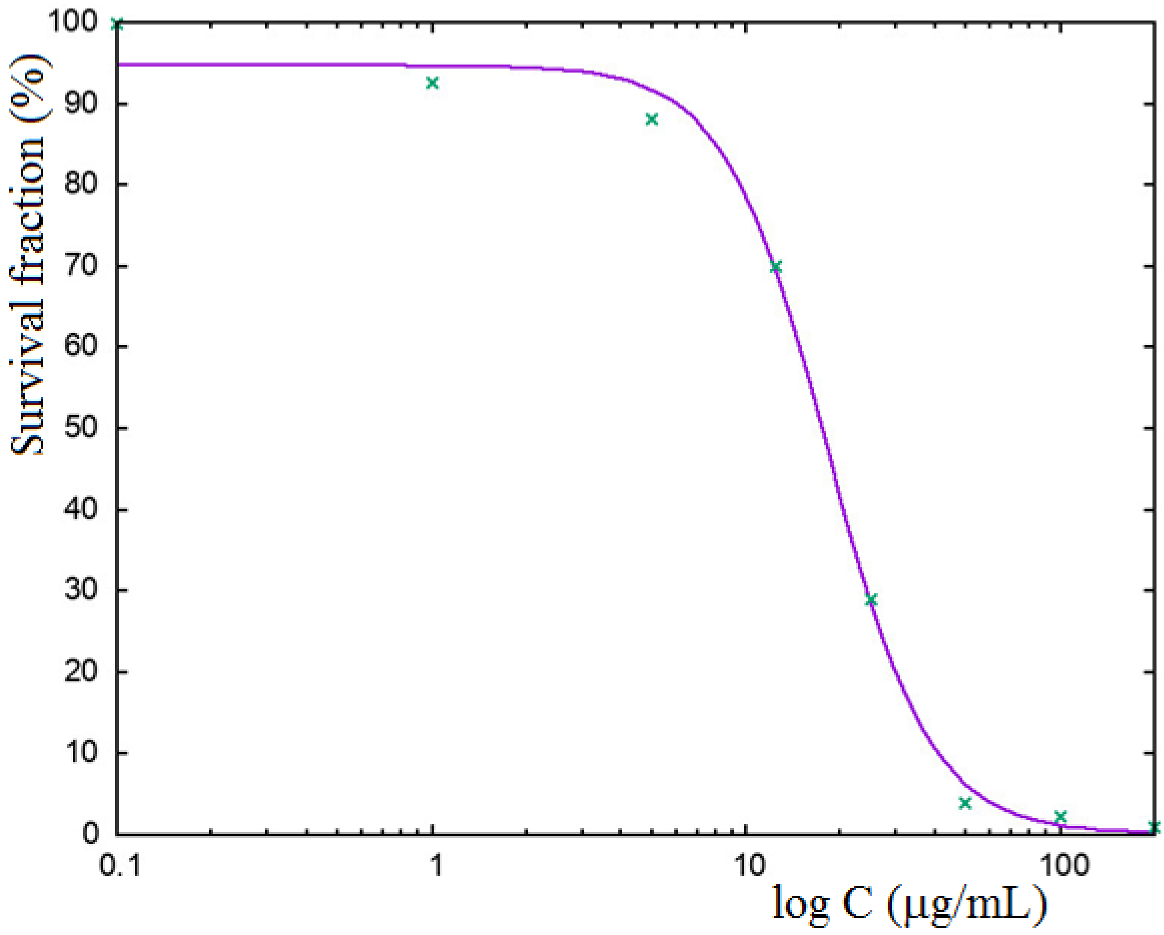

Figure S15. Dependence of A375 human cancer cell viability on concentrations of cisplatin.

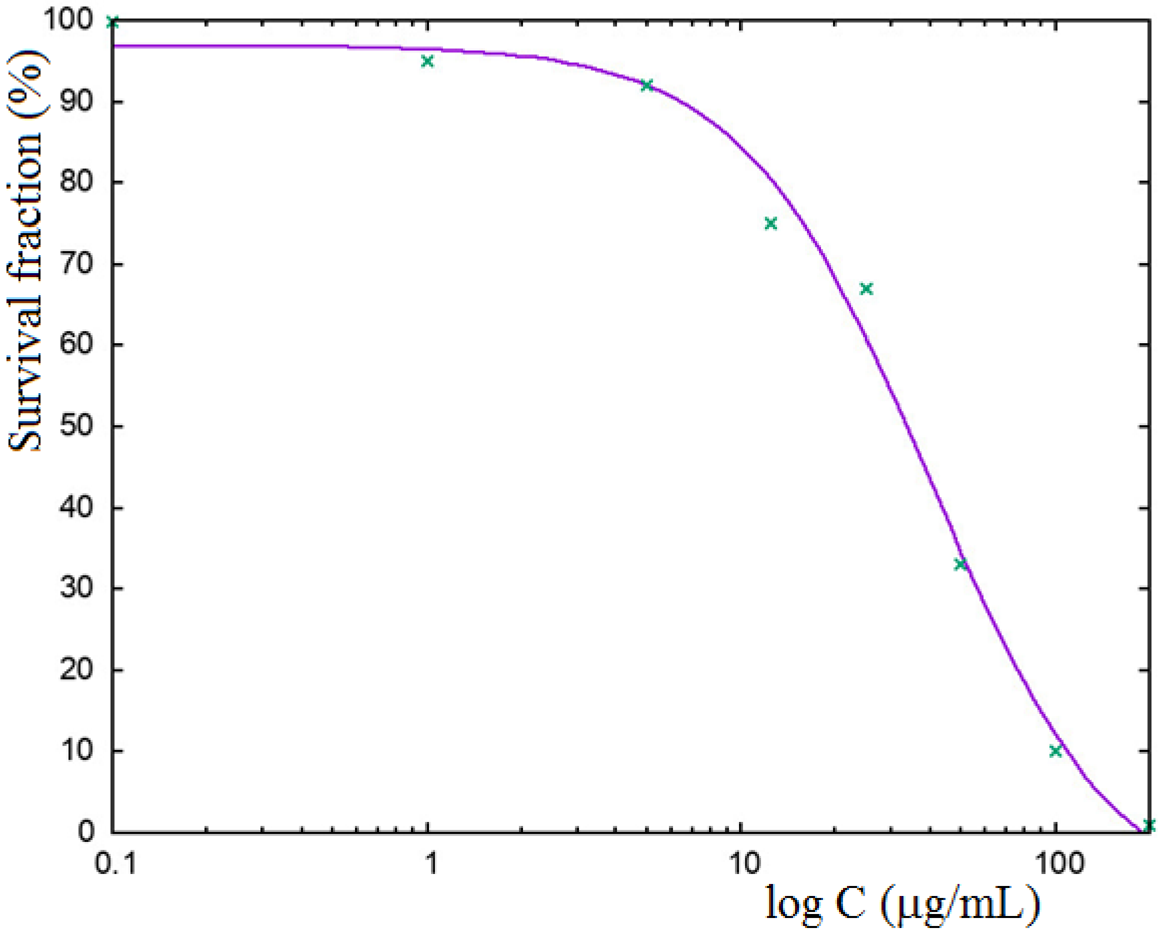

Figure S16. Dependence of WS1 human cancer cell viability on concentrations of cisplatin.

Table S1. Lipophilicity of alkaloid standards (PubChem, https://pubchem.ncbi.nlm.nih.gov/).

Name of alkaloid	Log P	Hydrogen bond acceptor count	Hydrogen bond donor count	Polar surface area (Å^2)
Berberine	3.6	4	0	40.8
Palmatine	3.7	4	0	40.8
Chelerythrine	4.6	4	0	40.8
Protopine	2.8	6	0	57.2
Sanguinarine	4.4	4	0	40.8
